# Biliary drainage in patients with malignant distal biliary obstruction: results of an Italian consensus conference

**DOI:** 10.1007/s00464-024-11245-4

**Published:** 2024-09-25

**Authors:** Marco Marzioni, Stefano Francesco Crinò, Andrea Lisotti, Lorenzo Fuccio, Giuseppe Vanella, Arnaldo Amato, Helga Bertani, Cecilia Binda, Chiara Coluccio, Edoardo Forti, Alessandro Fugazza, Dario Ligresti, Marcello Maida, Giovanni Marchegiani, Aurelio Mauro, Vincenzo Giorgio Mirante, Claudio Ricci, Giacomo Emanuele Maria Rizzo, Daniela Scimeca, Marco Spadaccini, Marianna Arvanitakis, Andrea Anderloni, Carlo Fabbri, Ilaria Tarantino, Paolo Giorgio Arcidiacono, i-EUS Group

**Affiliations:** 1 Clinic of Gastroenterology and Hepatology, Università Politecnica delle Marche – Azienda Ospedaliero Universitaria delle Marche, Ancona, Italy; 2 Diagnostic and Interventional Endoscopy of Pancreas, The Pancreas Institute, G.B. Rossi University Hospital, 37134 Verona, Italy; 3 Gastroenterology Unit, Hospital of Imola, University of Bologna, Imola, Italy; 4 Department of Medical and Surgical Sciences, University of Bologna - Gastroenterology Unit, IRCCS Azienda Ospedaliero-Universitaria di Bologna, S. Orsola Hospital, Bologna, Italy; 5 Pancreatobiliary Endoscopy and Endosonography Division, Pancreas Translational and Clinical Research Center, IRCCS San Raffaele Institute, Milan, Italy; 6 Department of Digestive Endoscopy and Gastroenterology ASST, Lecco, Italy; 7 Gastroenterologia ed Endoscopia Digestiva Azienda Ospedaliero-Universitaria Policlinico di Modena, Modena, Italy; 8 Gastroenterology and Digestive Endoscopy Unit, Forlì-Cesena Hospitals, AUSL Romagna, Forlì-Cesena, Italy; 9 Digestive and Interventional Endoscopy Unit, ASST Niguarda Hospital, Milan, Italy; 10 Division of Gastroenterology and Digestive Endoscopy, Humanitas Research Hospital - IRCCS, Rozzano, 20089 Milan, Italy; 11 Endoscopy Service, Department of Diagnostic and Therapeutic Services, IRCCS - ISMETT, Palermo, Italy; 12 Gastroenterology Unit, Umberto I Hospital - Department of Medicine and Surgery, University of Enna ‘Kore’, Enna, Italy; 13 Department of Surgical Oncological and Gastroenterological Sciences, Padua University Hospital, Padua, Italy; 14 Gastroenterology and Digestive Endoscopy Unit, IRCCS Foundation Policlinico San Matteo, Viale Camillo Golgi 19, 27100 Pavia, Italy; 15 Gastroenterologia ed Endoscopia Digestiva, Dipartimento Oncologico e Tecnologie Avanzate, AUSL IRCCS Reggio Emilia, Reggio Emilia, Italy; 16 Department of Medical and Surgical Sciences, University of Bologna - Division of Pancreatic Surgery, IRCCS Azienda Ospedaliero-Universitaria Di Bologna, Bologna, Italy; 17 Gastroenterology and Endoscopy Unit, ARNAS Civico - Di Cristina - Benfratelli Hospital, 90127 Palermo, Italy

**Keywords:** Malignant distal biliary obstruction, Cholangiocarcinoma, Pancreatic cancer, Endoscopic ultrasonography, Lumen apposing metal stent

## Abstract

**Background:**

Malignant Distal Biliary Obstruction (MBDO) is a common event occurring along the natural history of both pancreatic cancer and cholangiocarcinoma. Epidemiological and biological features make MBDO one of the key elements of the clinical management of patients suffering for of pancreatic cancer or cholangiocarcinoma. The development of dedicated biliary lumen-apposing metal stents (LAMS) is changing the clinical work up of patients with MBDO. i-EUS is an Italian network of clinicians and scientists with a special interest in biliopancreatic endoscopy, EUS in particular.

**Methods:**

The scientific methodology was chosen in line with international guidance and in a fashion similar to those applied by broader scientific associations. PICO questions were elaborated and subsequently voted by a broad panel of experts within a simplified Delphi process.

**Results and conclusions:**

The manuscripts describes the results of a consensus conference organized by i-EUS with the aim of providing an evidence based-guidance for the appropriate use of the techniques in patients with MBDO.

**Supplementary Information:**

The online version contains supplementary material available at 10.1007/s00464-024-11245-4.

Malignant Distal Biliary Obstruction (MBDO) is a common event occurring along the natural history of both pancreatic cancer and cholangiocarcinoma. Pancreatic cancer is a leading cause of death in particular in western countries, is global burden, and is rising over time, having doubled in the last two decades [1]. Cholangiocarcinoma is a rare and heterogeneous disease; its incidence and mortality rates have increased globally (0.3–6 cases per 100,000 inhabitants yearly in Western countries, and > 6 cases in some East Asian regions) over the past 20 years [2]. In addition to a similar epidemiological trend, pancreatic cancer and cholangiocarcinoma share aggressive biological features and high mortality rates.

Epidemiological and biological features make MBDO one of the key elements of the clinical management of patients suffering for pancreatic cancer or cholangiocarcinoma. Biliary drainage is determinant for the administration of oncological therapy and has a direct effect on patients’ quality of life [3]. The current gold standard technique to achieve biliary drainage is the transpapillary route by retrograde cholangiopancreatography (ERCP), with an alternative in Percutaneous Bile duct drainage (PTBD) in case of failure.

The development of dedicated biliary lumen-apposing metal stents (LAMS) is changing the clinical work-up of patients with MBDO. The use of LAMS simplifies the establishment of a choledochoduodenal anastomosis by Endoscopic Ultrasonography (EUS); its promising results convey the potential for a greater clinical application [3].

i-EUS is an Italian network of clinicians and scientists with a special interest in biliopancreatic endoscopy, EUS in particular. In the current phase of initial dissemination of application of EUS to the management of MBDO, i-EUS thought to organize a consensus conference with the aim of providing an evidence-based guidance for the appropriate use of the techniques that are being developed. In particular, the experts reviewed the current evidence on four main aspects of the clinical management of MBDO, to answer the following questions: (i) which are the indications to biliary drainage? (ii) which is the best approach to EUS-guided biliary drainage? (iii) how to manage specific challenges? (iv) how to take care of and follow up patients?

## Methods

The scientific methodology was chosen in line with international guidance and in a fashion similar to those applied by broader scientific associations [4], with pre-defined changes for the method to be applicable to the current set.

The process involved identification of a number of key questions pertinent to the subject matter. The expert panel drafted questions according to the PICO format (P—patient, problem or population, I—intervention, C—comparison, control or comparator, O—outcome). i-EUS identified 20 experts divided in 4 work groups who defined the PICO questions and discussed in three virtual meetings for approval. This was followed by a systematic literature review process, for statement definition and grading of the evidence. Search terms and results can be found in the supplementary information. The level of evidence was graded according to the Oxford Centre for Evidence-Based Medicine (OCEBM) system (Table 1) and the strength of the recommendations was categorized as either ‘weak’ or ‘strong’ (Table 2) [4]. The higher the quality of the evidence, the more likely a strong recommendation was made. If no clear evidence was available, recommendations were based on the expert opinion of the panel members. A simplified Delphi process followed: statements and evidences were presented in a dedicated, in-person plenary session (Palermo, Italy, May 2023), where results were discussed by a total of 61 experts (gastroenterologists/endoscopists, radiologists, surgeons). Experts were then called to vote for approval of final statements; a pre-defined cut off of 80% of consensus was considered valid for statement approval. The consensus reached for each PICO question is reported in Table 3.

**Table 1:** Level of evidence based on the Oxford Centre for Evidence-based Medicine (adapted)

Level	Criteria	Simple model for high, intermediate and low evidence
1	Systematic Reviews (SR) (with homogeneity) of Randomized controlled trials (RCT)	Further research is unlikely to change our confidence in the estimate of benefit and risk
2	Randomized controlled trials (RCT) or observational studies with dramatic effects; Systematic Reviews (SR) of lower quality studies (i.e., non-randomized, retrospective)	
3	Non-randomized controlled cohort/follow-up Study/control arm of randomized trial (systematic review is generally better than an individual study)	Further research (if performed) is likely to have an impact on our confidence in the estimate of benefit and risk and may change the estimate
4	Case-series, case–control, or historically controlled studies (systematic review is generally better than an individual study)	
5	Expert opinion (Mechanism-based Any estimate of effect is uncertain Reasoning)	Any estimate of effect is uncertain

**Table 2:** Grades of recommendation

Grade	Wording	Criteria
Strong	Shall, should, is recommended shall not, should not, is not recommended	Evidence, consistency of studies, risk- benefit ratio, patient preferences, ethical obligations, feasibility
Weak/open	Can, may, is suggested may not, is not suggested

**Table 3:** Agreement to the proposed statements

Statement	Agreement (%)
**Statement #1** **i-EUS group suggests EUS choledocho-duodenostomy (EUS-CDS) as an alternative to ERCP as first attempt for biliary drainage in patients with unresectable distal malignant biliary obstruction** *(Level of evidence: 2, grade of recommendation: strong)*	91.8
**Statement #2a** **i-EUS group recommends against routine preoperative biliary drainage in patients with resectable malignant distal biliary obstruction** *(Level of evidence: 1, grade of recommendation: strong)*	94.8
**Statement #2b** **i-EUS group recommends preoperative biliary drainage in patients with cholangitis, severe jaundice, in those planned for neoadjuvant therapy, and when a delay in surgery is anticipate** *(Level of evidence: 1, grade of recommendation: strong)*	96.4
**Statement #3** ***i-EUS group recommends EUS-guided choledochoduodenostomy (EUS-CDS) over PTBD after failed ERCP in malignant unresectable distal MDBO*** *(Level of evidence: 2, grade of recommendation: strong)*	93.2
**Statement #4** **i-EUS group suggests performing EUS-BD under deep sedation or general anesthesia; the choice between these two options should be based on patient clinical condition and anaesthesiologist preference** *(Level of evidence: 4, grade of recommendation: weak)*	91.3
**Statement #5** **i-EUS recommends EUS-choledocoduodenostomy for the treatment of MBDO after ERCP failure. EUS-guided gallbladder drainage (EUS-GBD) and EUS-guided hepaticogastrostomy (EUS-HGS) should be considered in case of unapproachable EUS-CDS** *(Level of evidence 2; Grade of recommendation: strong)*	92
**Statement #6** **i-EUS group states that EUS-guided gallbladder drainage (EUS-GBD) may be considered a rescue strategy for biliary drainage in unresectable patients with MBDO and patent cystic duct when other EUS-BD approaches are not feasible** *(Level of evidence 3; Grade of recommendation: weak)*	84.7
**Statement #7a** **iEUS recommends the use of either self-expandable metal stents (SEMS) or lumen-apposing metal stents (LAMS) for EUS-guided choledochoduodenostomy (EUS-CDS)** *(Level of evidence 3; Grade of recommendation: strong)*	93
**Statement #7b** **iEUS suggests that of electrocautery enhanced LAMS might be preferred in patients with dilated (≥ 15 mm) common bile duct since a slight reduction in pooled incidence of adverse events was observed** *(Level of evidence 3; Grade of recommendation: weak)*	91.3
**Statement #8** **I-EUS does not suggest the use of 6 × 8 mm over 8 × 8 mm LAMS since no advantages has been demonstrated; 6 × 8 mm LAMS could be used in case of small CBD diameter** *(Level of evidence 4; Grade of recommendation: weak)*	84.4
**Statement #9** **There is no evidence to suggest in favor or against double pig-tail plastic stents (DPPS) placement through the LAMS in patients undergoing EUS-guided choledochoduodenostomy (EUS-CDS). i-EUS states that DPPS placement could be considered in selected cases** *(Level of evidence 4; Grade of recommendation: weak)*	98.2
**Statement #10** **i-EUS suggests the use of dedicated stents in patients undergoing EUS-guided hepaticogastrostomy (EUS-HGS) for MBDO** *(Level of evidence 5, Grade of recommendation: open)*	94.7
**Statement #11** **i-EUS suggests that EUS-gastroenterostomy (EUS-GE) may be preferred over Enteral Stenting in patients with malignant Gastric Outlet Obstruction** (*Level of evidence 3, Grade of recommendation: weak*)	91.3
**Statement #12a** **i-EUS suggests that, in the setting of double obstruction, ERCP may be attempted whenever the papilla is reachable (especially in type 1 or 3 stenosis) or in case of previously placed duodenal stent** (*Level of evidence 4, Grade of recommendation: weak*)	91.5
**Statement #12b** **i-EUS suggests that, in naïve patients with double obstruction, EUS-guided double bypass may be considered where adequate expertise is available** (*Level of evidence 4, Grade of recommendation: weak*)	91.5
**Statement #13** **i-EUS suggests that, in the specific setting of double obstruction, EUS-guided hepaticogastrostomy (EUS-HGS) may be favored over EUS-guided choledochoduodenostomy (EUS-CDS) due to longer stent patency** (*Level of evidence 4, Grade of recommendation: weak*)	85.9
**Statement #14** **i-EUS suggests either ERCP or EUS-guided choledochoduodenostomy (EUS-CDS) as the first-line treatment of resectable MBDO** (*Level of evidence 4, Grade of recommendation: weak*)	85
**Statement 15a** **In patient with malignant distal biliary stenosis and surgical altered anatomy, treatment choice depends on disease extension and type of reconstruction. i-EUS suggests to consider EUS-BD over laparoscopic-/enteroscopy-assisted ERCP and PTBD in patient with Roux-en-Y anatomy** (*Level of evidence 4, Grade of recommendation: weak*)	85
**Statement 15b** **i-EUS suggests to consider EUS-BD as a rescue strategy after failed ERCP with lateral-viewing or cap-assisted frontal-viewing endoscopes in patients with Billroth II reconstruction** (*Level of evidence 4, Grade of recommendation: weak*)	83.3
**Statement #16** **I-EUS suggests multidisciplinary discussion of patients with distal malignant obstruction in whom the biliary drainage could impact on the main outcomes, in particular patients with resectable cancer, altered anatomy, and double obstruction** (*Level of evidence 5, Grade of recommendation: weak*)	89
**Statement #17** **I-EUS suggests endoscopic biliary drainage to be performed in a setting, where adequate competencies in interventional bilio-pancreatic management are available** (*Level of evidence 5, Grade of recommendation: weak*)	92.9
**Statement #18** **i-EUS does not suggest any specific diet after EUS-guided biliary drainage to prevent stent dysfunction** (*Level of evidence 5, Grade of recommendation: weak*)	91.2
**Statement #19** **i-EUS does not suggest the routine use of antibiotic prophylaxis, to reduce the risk of post-procedural complications after EUS-guided biliary drainage** **i-EUS suggest that antibiotic prophylaxis should be offered in selected patients (e.g., immunocompromised patients, expected incomplete biliary drainage)** (*Level of evidence 5, Grade of recommendation: weak*)	92.9
**Statement #20** **i-EUS does not suggest the administration of medical therapy to improve endoscopic outcomes after biliary drainage for malignant distal biliary obstructions** **iEUS does not suggest the use of ursodeoxycholic acid (UDCA) since it is not effective in preventing recurrent biliary obstruction after SEMS placement and may increase the risk of stent occlusion** (*Level of evidence 5, Grade of recommendation: weak*)	98.2
**Statement #21** **i-EUS suggests obtaining a “goal-based” informed consent for endoscopic malignant biliary drainage prior to either EUS-guided drainage or conventional ERCP** *(Level of evidence: 5; Grade of recommendation: weak)*	92.8

**WHICH ARE THE INDICATIONS TO BILIARY DRAINAGE?**

**CLINICAL QUESTION #1**

**Can Endoscopic ultrasound biliary drainage (EUS-BD) be considered as first option for biliary drainage of malignant distal biliary obstruction?**

**Statement #1**

**i-EUS group suggests EUS choledocho-duodenostomy (EUS-CDS) as an alternative to ERCP as first attempt for biliary drainage in patients with unresectable distal malignant biliary obstruction.**

*(Level of evidence: 2, grade of recommendation: strong)*

**Summary of evidence**

ERCP is currently considered the first-line approach to obtain adequate biliary drainage. EUS-BD has been developed in the last two decades to perform a direct communication between the biliary tree and the gastroduodenal lumen when ERCP fails or is not feasible. EUS-BD could be performed with different techniques, the two more relevant are CDS connecting the common bile duct and the duodenal lumen, and hepaticogastrostomy (HGS) connecting the left intrahepatic segments and the gastric lumen. Both CDS and HGS have demonstrated high technical feasibility, clinical success, and safety after failed ERCP.

Four Randomized Controlled Trials (RCT) have evaluated the efficacy and safety of EUS-BD as a primary treatment in patients with distal malignant biliary obstruction (DMBO) compared to ERCP [5–8]. EUS-BD was performed via CDS in all but one the studies where HGS was used in 50% of patients allocated in the EUS-BD arm [6].

In three RCT [6–8], only patients with unresectable DMBO were included, whereas in the study by Bang et al., a minority of resectable patients were also included [5]. In the first three RCTs, EUS-BD was performed using a self-expandable metal stent (SEMS). Differently, in the RCT by Teoh et al., hot-LAMS were used. All the four RCT showed similar results between EUS-BD and ERCP in terms of technical and clinical success and safety. In the last study by Teoh et al. technical success was higher in the EUS-BD group (96.2% vs 76.3%, *p* < 0.001) but it should be noted that technical failure in the ERCP group was related to duodenal obstruction in 7 patients [8]. Comparable efficacy and safety of EUS-BD and ERCP were confirmed in different meta-analysis [9, 10]. In a more recent RCT, EUS-CDS with (hot) LAMS demonstrated to be noninferior to ERCP in terms of technical success (90.4% vs 83.1%) with comparable stent dysfunction rate at 1 year (9.6% vs 9.9%), safety (adverse events rate of 12.3% vs 12.7%), and initial clinical success (84.9% vs 85.9%) [11].

**CLINICAL QUESTION #2**

**Is biliary drainage indicated in patients with resectable malignant distal biliary obstruction?**

**Statement #2a**

**i-EUS group recommends against routine preoperative biliary drainage in patients with resectable malignant distal biliary obstruction.**

*(Level of evidence: 1, grade of recommendation: strong)*

**Statement #2b**

**i-EUS group recommends preoperative biliary drainage in patients with cholangitis, severe jaundice, in those planned for neoadjuvant therapy, and when a delay in surgery is anticipate.**

*(Level of evidence: 1, grade of recommendation: strong)*

**Summary of evidence**

Persistent cholestasis due to distal malignant biliary obstruction (DMBO) is associated with post-surgical complications, including anastomotic leaks and poor wound healing. On this basis, preoperative biliary drainage (PBD) has been used to reduce surgical morbidity and mortality in patients with jaundice undergoing pancreaticoduodenectomy for DMBO. However, conflicting data about the usefulness of PBD have been reported. In 2010, a multicenter RCT provided additional data on this topic [12]. A total of 202 patients with resectable pancreatic head cancer were randomized to early surgery within 1 week without PBD or to ERCP with PBD and delayed surgery 4–6 weeks later. The rate of cumulative post-surgical serious complication was significantly higher in the PBD group (mostly due to the PDB procedure, as cholangitis or stent occlusion) compared to the early surgery group (39% vs. 74%, *p* < 0.001) with a relative risk of complications after early surgery of 0.54 (95% CI 0.41–0.71). Mortality and length of hospital stay did not differ significantly between the two groups.

Multiple meta-analyses, including retrospective and prospective studies and RCTs, assessed the potential benefit of PBD in patients with distal MDBO. None of them found differences in terms of mortality. In terms of morbidity, only one meta-analysis [13] reported a lower rate of serious adverse events in patients who underwent PBD, whereas other meta-analyses found similar [14, 15] or higher [16, 17] morbidity in patients with PBD vs those without PBD, with regard to infection and cholangitis rates. Consequently, European guidelines did not recommend PBD in all patients with MDBO [18].

However, specific settings of patients requiring PBD were identified, such as acute cholangitis and severe jaundice (with or without symptoms). Indeed, acute cholangitis represents an urgent condition requiring prompt intervention, and severe jaundice was associated with a higher risk of postoperative complications. For this reason, bilirubin levels > 15 mg/dl are considered an indication for PBD, especially when a delay in surgery is anticipated.

Moreover, PBD is required in patients with obstructive jaundice planned to receive neoadjuvant chemotherapy and could be indicated to improve alterations in coagulation and fibrinolysis related to jaundice.

**CLINICAL QUESTION #3**

***How biliary drainage should be performed in non-surgical candidate patients with malignant distal biliary obstruction when ERCP fails?***

**Statement #3**

***i-EUS group recommends EUS-guided choledochoduodenostomy (EUS-CDS) over PTBD after failed ERCP in malignant unresectable MBDO.***

*(Level of evidence: 2, grade of recommendation: strong)*

**Summary of evidence**

Difficult biliary cannulation during ERCP (which accounts for up to 10%) is associated with a higher risk of ERCP-related complications [5]. PTBD has historically been considered a rescue strategy in case of ERCP failure, despite it is burdened by significant morbidity and complications. Many recent retrospective and prospective studies demonstrated the efficacy (technical and clinical success of 90–98%), and safety (AE rate of 5–23%) of EUS-BD performed with LAMS as a second option after failed ERCP [6, 7].

According to the growing evidence, Asian and European guidelines recommended EUS-BD as the procedure of choice for biliary drainage in patients with failed ERCP [8, 9] when local expertise is available. These recommendations are supported by available evidence coming from 28 retrospective and 8 prospective studies, 4 randomized controlled trials (RCTs), and 8 meta-analyses, which compared EUS-BD and PTBD in patients with MBDO reporting similar technical and clinical success rates for EUS-BD versus percutaneous or surgical treatment, with lower rate of AE and reintervention, even resulting in better cost-effectiveness (Supplementary Table 3). It should be noted that the examined population is not homogeneous throughout studies because it is not always specified if only patients with unresectable diseases were included and, in addition, in most of the studies the subgroup of EUS-BD is characterized by one or more of the different techniques available.

Another option in case of ERCP failure, especially when expertise for EUS-guided techniques is not available or dilation of biliary system is not enough to safely perform it, can be represented by repeating ERCP on a different timing (interval ERCP). This option should be considered mainly if a precut has been performed at the index procedure. Available studies, including benign and malignant indications, examined the benefit of interval ERCP reporting a successful cannulation rate ranging between 68 and 87%, with an AE rate comparable to index ERCP [10, 12, 13]. Also, percutaneous rendezvous technique remains a potential option with a successful cannulation rate of approximately 90% [14].

**CLINICAL QUESTION #4**

**Which type of sedation should be preferred for EUS-BD in malignant distal biliary obstruction?**

**Statement #4**

**i-EUS group suggests performing EUS-BD under deep sedation or general anesthesia; the choice between these two options should be based on patient clinical condition and anaesthesiologist preference.**

*(Level of evidence: 4, grade of recommendation: weak)*

**Summary of evidence**

No studies comparing different sedation/anesthesia strategies for EUS-BD in patients with MBDO have been published. In available studies, EUS-BD (including choledochoduodenostomy and hepaticogastrostomy) has been performed either under conscious sedation, deep sedation, and general anesthesia, with the latter appearing to be the most used [6, 19]. A recent Italian survey on LAMS clinical practice confirmed this variability in sedation preference: general anesthesia was used in 38.8% of the centers involved (14/36), deep sedation in 30.5% (11/36), and conscious sedation in 8.3% (3/36) [20]. This issue has been raised also by the ESGE Technical Review on Therapeutic EUS, which stated that although many centers perform therapeutic EUS exclusively in patients under general anesthesia, other centers use conscious or deep sedation without compromising safety outcomes [21]. Likewise, ERCP biliary drainage can be performed under conscious or deep sedation or general anesthesia, and the choice of type of sedation/anesthesia is often based on local resources and institutional preferences [22]. However, a Dutch study showed that one-third to one-half of patients experienced pain and discomfort during and immediately after ERCP when the procedure is performed under conscious sedation [23]. Similarly, it is likely that conscious sedation can be a burden for the patient undergoing EUS-BD and, therefore, as with ERCP, other forms of deeper sedation should be preferred.

**WHICH IS THE BEST APPROACH TO EUS-GUIDED BILIARY DRAINAGE?**

**CLINICAL QUESTION #5**

**What’s the best method of EUS-guided biliary drainage in terms of technical and clinical success, safety and long-term patency among patients with malignant distal biliary obstruction after ERCP failure?**

**Statement #5**

**i-EUS recommends EUS-choledocoduodenostomy for the treatment of MBDO after ERCP failure. EUS-guided gallbladder drainage (EUS-GBD) and EUS-guided hepaticogastrostomy (EUS-HGS) should be considered in case of unapproachable EUS-CDS.**

*(Level of evidence 2; Grade of recommendation: strong)*

**Summary of evidence**

EUS-guided BD has been an effective alternative method to PTBD after ERCP failure for DMBO. EUS-guided biliary drainage may include different approaches, as choledocoduodenostomy (CDS), hepaticogastrostomy (HGS), antegrade stenting, rendezvous or even gallbladder drainage, but strong outcomes comparisons are still lacking in the literature in the setting of patients with MBDO after ERCP failure. Recently, a network meta-analysis including 5 RCTs (217 patients) evaluated those methods for BD of MBDO after ERCP failure in terms of technical success, clinical success, and postprocedure adverse events. In this study, EUS-BD included EUS-CDS and EUS-HGS, which showed no significant differences when compared each other (EUS-HGS vs EUS-CDS: clinical success RR 1.01, 95% CI 0.87–1.17; technical success RR 1.01, 95% CI 0.93–1.09; adverse event rate RR 0.85, 95% CI 0.39–1.82) [24]. Our literature search analyzed 28 studies. The majority of studies were retrospective (*n* = 18, 62%, Table 1), while other studies included four trials (Supplementary Table 5) [25–28] and six prospective studies (Table 3). Fifteen studies were multi-center (53.6%). However, there was no direct efficacy and safety comparison among EUS-BD procedures, even if eight studies included two or more different EUS-guided BD procedures. Pooled technical and clinical success of EUS-BD was over 95% when considering all of the approaches, with a low heterogeneity among studies even if not statistically significant (TS, *I*^2^ = 5.03%, *p* = 0.379; CS, *I*^2^ = 25.38%, *p* = 0.083). Pooled technical success for EUS-CDS and EUS-HGS was, respectively, 95.5% and 94.7%, while clinical success was 96.5% and 90.9%, respectively. Furthermore, EUS-GBD after ERCP failure was used when EUS-CDS was unapproachable among the included studies, showing a pooled technical and clinical success of 97.7% and 92.4%, respectively. In this setting of patients, EUS-guided rendezvous (EUS-RV) was used in two studies (*n* = 15 patients) showing 100% of clinical and technical success, while the EUS-guided antegrade (EUS-AG) approach was used for 40 patients (two studies) with a technical success of 97.4% (*n* = 38) and 100% (*n* = 2), but clinical success (100%, 2 out of 2 patients) was reported only in one study. Moreover, EUS-CDS showed better safety compared to EUS-HGS and EUS-GBD (CDS 10.3% vs HGS 16.8% vs GBD 15.5%). However, some study also showed data on patency, with overall mean patency rate of 69.4% (± 24.98) during the period of follow-up.

**CLINICAL QUESTION #6**

**Could EUS-GBD be considered as a rescue strategy in patients with malignant distal biliary obstruction and patent cystic duct when other EUS-BD approaches are not feasible?**

**Statement #6**

**i-EUS group states that EUS-guided gallbladder drainage (EUS-GBD) may be considered a rescue strategy for biliary drainage in unresectable patients with MBDO and patent cystic duct when other EUS-BD approaches are not feasible.**

*(Level of evidence 3; Grade of recommendation: weak)*

**Summary of evidence**

To date, EUS-guided drainages are a valuable alternative in case of failure of ERCP in patients affected by DMBO, usually when are considered unfit for surgery. Although EUS-BD has a high technical success rate and an acceptable risk profile, EUS-BD can fail or may be technically unfeasible for multiple reasons, such as in case of a CBD < 15 mm or altered anatomy. EUS-guided gallbladder drainage (EUS-GBD) is a feasible rescue therapy when ERCP and EUS-BD are unsuccessful or not feasible, which can occur in up to 7% of patients with DMBO. In 2016, Imai et al. reported a series of 12 cases with malignant biliary obstruction who underwent EUS‑GBD after failed ERCP and EUS‑BD [29]. The rational is that the bile produced by the liver, through the cystic duct passed usually goes in the gallbladder and then is again excreted in the bile duct. Therefore, when a DMBO is present, the gallbladder can represent a possible gate for drainage. However, in order to perform an EUS-GBD as rescue treatment, the patency of the cystic duct is often not easy to be assessed and should be carefully evaluated at CT-scan and during EUS examination. To date, 3 case reports and 5 retrospective studies are available with at least 116 patients (see Table 1). In 6 studies, the authors used LAMS for EUS-GBD, while in the remaining, 2 studies SEMS were used.

A recent meta-analysis reported a pooled technical success of 100%, pooled clinical success of 85% and a pooled rate of adverse events of 13%, being stent dysfunction the most common complication [30]. The pooled rate of stent dysfunction was 9%, mainly due to food impaction in the stent complicated by recurrent cholecystitis and to the entrapment of the cystic duct by growing tumor [30]. Three studies report the mean percentage of decrease in bilirubin levels at 14 days with EUS-GBD, which ranges from 62 to 66.5% [5, 9, 10]. In most of the cases, patients were considered unresectable, while only in few cases, a subsequent surgery was performed. Therefore, as evidence are sparse regarding this procedure in the setting of EUS-GBD as a bridge-to-surgery, we suggest to reserve this type of drainage in the setting of unresectable diseases.

**CLINICAL QUESTION #7**

**In patients with malignant distal biliary obstruction undergoing EUS-guided choledochoduodenostomy (EUS-CDS), is there any advantage in the use of either LAMS or SEMS?**

**Statement #7a**

**iEUS recommends the use of either self-expandable metal stents (SEMS) or lumen-apposing metal stents (LAMS) for EUS-guided choledochoduodenostomy (EUS-CDS).**

*(Level of evidence 3; Grade of recommendation: strong)*

**Statement #7b**

**iEUS suggests that of electrocautery enhanced LAMS might be preferred in patients with dilated (≥ 15 mm) **common** bile duct since a slight reduction in pooled incidence of adverse events was observed.**

*(Level of evidence 3; Grade of recommendation: weak)*

**Summary of evidence**

In terms of comparison of the different stent types for EUS-CDS, a systematic review conducted in 2021 [31] identified 31 studies including 820 patients who underwent EUS-CDS with either SEMS (25 studies; 509 patients) or LAMS (6 studies; 311 patients). The authors did not identify significant difference in terms of technical success rate (92.7% vs. 94.8%), clinical success rate (91.7% vs 93.6%), adverse event rate (18.3% vs. 17.1%), and reintervention rate (13.9% vs. 10.9%).

One retrospective study [32] compared the outcomes of 20 patients who underwent EUS-CDS with SEMS to 37 patients who underwent EUS-CDS with LAMS. The authors reported similar rates of technical success (100% in both groups), clinical success (95% vs. 100%), adverse event (20% vs. 13.5%), and reintervention rate (35% vs. 16.2%). Finally, overall survival was similar in the two groups.

A systematic review updated at the end of April 2023 identified thirty-eight original studies including 1277 patients who underwent EUS-CDS for distal MBO (Supplementary Table 6). The above-mentioned retrospective comparative study on SEMS vs. LAMS, 26 (7 RCTs, 9 prospective and 10 retrospective) studies using SEMS and 12 (1 prospective, 11 retrospective) studies using LAMS. Finally, 570 patients underwent EUS-CDS with SEMS, while 707 with LAMS. Pooled outcomes of EUS-CDS assessed with random effect model are summarized in (Supplementary Table 7).

Overall, the quality of available literature on the comparison between LAMS or SEMS use for EUS-CDS was low or even very-low. No randomized controlled trial designed to compare EUS-CDS with LAMS or SEMS.

**CLINICAL QUESTION #8**

**In patients with distal malignant distal biliary obstruction undergoing EUS-guided choledochoduodenostomy (EUS-CDS), the use of 6 mm LAMS allows advantages over ≥ 8 mm LAMS?**

**Statement #8**

**I-EUS does not suggest the use of 6 × 8 mm over 8 × 8 mm LAMS since no advantages has been demonstrated; 6 × 8 mm LAMS could be used in case of small CBD diameter.**

*(Level of evidence 4; Grade of recommendation: weak)*

**Summary of evidence**

There are several different LAMS systems available at this time with different lengths and diameters.

The major data of the EUS-CDS studies present in literature concern the Hot-Axios system (Boston Scientific, Massachusetts, USA).

In particular, 4 studies report data regarding 6–8 vs 8–8 mm stents. Jacques et al. reported that, on univariate analysis, the use of 6 × 8 mm LAMS (OR 6.67; P = 0.04) was related to an increased technical success [33].

In a prospective study, the same authors enrolled 70 patients and recommend to use a 6–8 mm stent based on their long experience with this stent size, without direct comparison of the impact of stent size on procedural outcomes [34].

In the largest multicentric study present in literature including 256 patients, no statistically significant difference was observed between 6 and 8 mm and 8–8 mm stents in terms of technical success, clinical success, and stent patency (*p* = 0.661) [19].

However, On et al. [35] in a multicentric retrospective study including 120 patients reported that the overall technical success was similar between patients who had a 6–8 mm and an 8–8 mm stent, but adverse events (OR, 3.71; 95% CI, 1.35–10.19; *p* = 0.008) and reintervention rates (OR, 6.17; 95% CI, 1.22–31.22; p = 0.019) were higher in those who had a 6-8 mm stent.

**CLINICAL QUESTION #9**

**In patients with malignant distal biliary obstruction undergoing EUS-guided choledochoduodenostomy (EUS-CDS) with LAMS (any size) the use of co-axial double pig-tail plastic stents (DPPS) allows any advantage?**

**Statement #9**

**There is no evidence to suggest in favor or against double pig-tail plastic stents (DPPS) placement through the LAMS in patients undergoing EUS-guided choledochoduodenostomy (EUS-CDS). i-EUS states that DPPS placement could be considered in selected cases.**

*(Level of evidence 4; Grade of recommendation: weak)*

**Summary of evidence**

LAMS obstruction could occur after EUS-CDS by food impaction from 6,7% to 31,8% of cases [19].

Placement of a double pig-tail stent (DPS) into a LAMS for EUS-CDS may be theoretical beneficial in preventing stent occlusion by maintaining patency of the lumen of the LAMS, anchoring the EC-LAMS to prevent migration, and orientating the LAMS in a vertical direction to augment coaxial biliary drainage [35].

In such clinical scenario, it can be considered to place a prophylactic DPPS through the internal lumen of the LAMS at the time of initial deployment.

El Chafic et al. [36] reported the first routine use of the double stent placement technique after placement of a 10–10 mm stent. In their cohort of 67 patients, 50 patients received double stent placement (DPS in 46 patients and Fully Covered SEMSs in 4 patients). Among 40 patients with follow-up of > 4 weeks, a significant reduction in biliary reintervention rates was observed in patients with double stent placement (5% vs 50%, *p* = 0 0.02).

In the multicenter retrospective study of On et al. [35], 32 patients placed DPPS into the LAMS. In this group of patients compared with patients who had only a LAMS were observed lower biliary reintervention (0% vs 12.2%, p = 0.03).

Non-statistically difference was encountered in the rate of cholangitis (6.3% vs 12.2%, *p* = 0.3).

Garcia-Sumalla et al. [37] published the first study assessing the hypothetical benefit of a DPS through a LAMS in EUS-CDS. In this retrospective study, 29 patients were included (22 LAMS alone vs 17 LAMS plus DPS). No differences between the groups in terms of clinical success (77.3 vs 87.5%, *p* = 0.67), adverse events (AEs, 13.6 vs 11.8%, *p* = 0.99), recurrent biliary obstruction (13.6 vs 23.5%, *p* = 0.67), or survival rate (*p* = 0.67) were encountered. The only difference was the procedural time: the LAMS alone group had a shorter length of procedure in comparison of the DPS group (50 min vs 66 min, *p* = 0.102). The authors conclude that a DPS inserted through a LAMS for EUS-CDS seems not to offer enough benefits over a LAMS alone, and it is a time-consuming approach. These results do not support this approach being used routinely.

**CLINICAL QUESTION #10**

**In patients undergoing EUS-guided hepaticogastrostomy (EUS-HGS) for malignant distal biliary obstruction, the use of dedicated stents (partially covered stents with uncovered distal portion, covered proximal portion, anti-migration flange/flap) allows any advantage over the use of other stents (fully covered SEMS, uncovered SEMS with a fully covered SEMS inside, DPPS)?**

**Statement #10**

**i-EUS suggests the use of dedicated stents in patients undergoing EUS-guided hepaticogastrostomy (EUS-HGS) for MBDO.**

*(Level of evidence 5, Grade of recommendation: open)*

**Summary of evidence**

EUS-HGS is characterized by a high technical complexity, that requires high technical skills both in ERCP and in interventional endoscopy, and not negligible rate of adverse events, often severe and requiring additional modalities (e.g., interventional radiology or surgery). Moreover, for a long time, no dedicated devices were available, with the employment mainly of plastic stents (PS), partially and fully covered self-expandable metal stents (SEMS). In the last years, different type of stent has been designed for this procedure, to make this procedure safer and to overcome the limitations of the previously available devices.

These stents are SEMS with a longer portion fully covered and uncovered extremity for the intra-hepatic side, and provided of variable anti-migrating systems both on the intra-hepatic side and the intra-gastric one [25, 38]. Therefore, these devices could potentially reduce the risk of both proximal and distal stent migration, biliary leakage, and intra-hepatic bile duct obstruction. There are no studies which directly compared the use of dedicated stents to non-dedicated ones. Standing to available literature, EUS-HGS outcomes varied widely among studies: technical success varies from 65 to 100%, clinical success from 76 to 100%, and overall, from 0 to 35% [25, 38–40].

To date the use of dedicated stents was reported in 20 studies on EUS-HGS, mainly mixed with non-dedicated stents and only in few cases considered alone. Standing to largest studies considering dedicated stents alone, technical success varies from 90.2 to 100%, clinical success from 83 to 100%, and adverse events from 9 to 25%. Interestingly, when considering the type of complications, transient fever, abscess, and sepsis were the most common adverse events, while no cases of biliary leakage were reported and only two cases of stent migration were described. One of the most severe adverse events after EUS-HGS is the occurrence of stent migration with consequent bile leak into the abdominal cavity. Dedicated stents have been specifically designed to avoid, or at least reduce, the risk of proximal migration, thanks to the presence of an uncovered portion that fixes the stent to the liver parenchyma. On the other hand, to reduce the risk of distal migration, choosing a stent length that guarantees a 2–3 cm margin into the gastric cavity is commonly suggested. While no data are available on the outcomes of EUS-HGS performed with different stent lengths, a recent meta-analysis demonstrated that dedicated stents significantly reduce the incidence of AEs after EUS-HGS (odd ratio 0.62) [41].

Although ESGE guidelines recommend either partially or fully covered SEMS [42], several expert opinions advised for the use of dedicated stents to reduce adverse events, especially stent migration.

**HOW TO MANAGE SPECIFIC CHALLENGES?**

**CLINICAL QUESTION #11**

**In the setting of malignant gastric outlet obstruction, should EUS-gastroenterostomy (EUS-GE) be preferred over enteral stenting?**

**Statement #11**

**i-EUS suggests to prefer EUS-gastroenterostomy (EUS-GE) over Enteral Stenting in patients with malignant Gastric Outlet Obstruction**

(*Level of evidence 3, Grade of recommendation: weak*).

**Summary of evidence**

Malignant gastric outlet obstruction (GOO) is common in pancreatobiliary and gastroduodenal malignancies, occurring in about 20% of patients [43]. Malignant GOO is thus associated with biliary obstruction (BO) in 40 to 92% of patients [44]. The goal of treatment is to palliate obstructive symptoms and to improve poor performance status that can delay chemotherapy treatment.

In the last decades, enteral stenting (ES) has been preferred over surgical gastroenterostomy as a treatment of GOO, given its noninvasive nature, high technical, clinical success, and safety profile. However, the main drawback of ES is represented by high recurrence rate reaching 56% at 6 months due to stent dysfunction related to ingrowth and overgrowth [45].

EUS-guided gastroenterostomy (EUS-GE) has recently emerged as a valuable alternative to enteral stenting, facilitating the effective reestablishment of the gastrointestinal transit while avoiding recurrences over time. Cohort comparative studies have demonstrated higher clinical success (ranging from 83.3 to 100% vs.67.3–87.7%) and lower recurrence rate (ranging from 1 to 8.8% vs. 26 to 33.3%) of EUS-GE over enteral stenting [11, 21, 46–49]. Technical success is reported to be the same or slightly inferior for EUS-GE but statistical significance was not reached.

Longer patency of EUS-GE with LAMS compared to ES seems to be related to the avoidance of the tumor tract reflecting a lower need for reintervention and a positive impact on the quality of life of the patient.

Also safety profile seems to be acceptable for EUS-GE with comparable adverse event rate with ES (7.1–16.7% vs. 10.3 vs. 40.2%) that however has been decreasing over time probably reflecting an improvement of the technique together with learning curve progression.

Results have been confirmed by two meta-analysis that reported higher clinical success, fewer severe adverse events, and lower GOO recurrence for EUS-GE [50, 51].

In the published series emerges that EUS-GE should not be performed in patients with significant ascites, peritoneal carcinomatosis, linitis plastica or previous gastric surgery.

It is to be considered that in the abovementioned available comparative studies, different EUS-GE techniques have been described comprising EPASS (EUS-guided balloon-occluded gastrojejunostomy bypass), direct unassisted EUS-GE with the water irrigation technique, direct puncture technique, balloon-assisted or nasobiliary catheter-assisted approach. This may have an impact on heterogeneity of results and the optimal method is yet to be confirmed.

Furthermore, being EUS-GE a technically challenging procedure even for the experienced endoscopist, it should be performed in tertiary care centers where a multidisciplinary expertise is available also to manage eventual adverse events.

Given the above and balancing risks and benefits, enteral stenting may be preferred in patients with a life expectancy less than 3 months.

**CLINICAL QUESTION #12**

**In the setting of concomitant biliary and gastric outlet malignant obstruction, what is the best modality to achieve biliary drainage?**

**Statement #12a**

**i-EUS suggests that, in the setting of double obstruction, ERCP may be attempted whenever the papilla is reachable (especially in type 1 or 3 stenosis) or in case of previously placed duodenal stent.**

(*Level of evidence 4, Grade of recommendation: weak*).

**Statement #12b**

**i-EUS suggests that, in naïve patients with double obstruction, EUS-guided double bypass may be considered where adequate expertise is available.**

(*Level of evidence 4, Grade of recommendation: weak*).

**Summary of evidence**

Management of combined biliary and gastric outlet obstruction is a challenging scenario. A duodenal obstruction can often impede standard ERCP or even reduce the efficacy of a technical successful biliary drainage. In this scenario, management strategies might vary according to the location of the stenosis (above, at or below the papilla) and the sequence of obstruction (biliary first, concomitant, GOO first).

In general, placement of duodenal stents (DS) has been associated with a non-negligible risk of pancreatitis and biliary obstruction, and it is also an independent predictor of dysfunction among patients with a biliary SEMS in place; it has been speculated that this depends not only on a direct interference of the meshes, but also on an increased duodenobiliary reflux due to duodenal invasion and reduced duodenal peristalsis.

On the other hand, when a DS has been already placed, obtaining biliary drainage becomes challenging, due to increased difficulties in traversing the duodenal stent, identifying or accessing the papilla or placing a stent through the meshes. Variable results have been reported regarding successful biliary drainage in retrospective series, ranging between 34 and 93%, with the variability probably reflecting the typical selection bias of retrospective evaluations [52–55]. However, what is clear is that ERCP underperforms in the setting of duodenal obstruction and indwelled duodenal SEMS, especially in type 2 stenoses involving the papilla [52–55] EUS-BD is increasingly used as a rescue in this setting, where the only other alternative would be PTBD with its morbidity and mortality [54]. ERCP might maintain optimal outcomes in type 1 or type 3 stenosis (where papilla is accessible), and when previous sphincterotomy or biliary stenting have already been performed [54]. When ERCP has proved unfeasible, EUS-BD might be very helpful. In a recent retrospective study including 39 patients, transmural drainage through EUS-BD demonstrated higher technical success (95% versus 56.0%; *p* < 0.01) and higher clinical success (91% versus 52.0%; *p* = 0.01) compared to ERCP with similar invasiveness and similar long-term patency. Moreover, when EUS-BD (EUS-HGS) has been compared to ERCP or PTBD in this setting, a trend toward lower rate of complications and reinterventions for the EUS group has been reported [56].

Series exploring the role of EUS-BD when DS was in place, tried different solutions, as for example through antegrade stenting, LAMS placement through the meshes of ES or EUS-HGS.

Limitations of this evidence are the following: (1) most studies involve retrospective evaluation of outcomes, despite prospective databases; (2) most studies include small cohorts (most EUS-BD cohorts < 10); (3) most studies are non-comparative, showing the efficacy and safety of single combination of procedures; (4) biliary and GO obstruction are often non-contemporary, with biliary management often performed times before GOO onset; (5) different demographical (cancer type), anatomical (location of the stenosis), and technical variables (extrahepatic, either gallbladder or choledochal drainage, versus intrahepatic EUS-BD; covered versus uncovered DS) are not controlled or corrected.

All these limitations emerge also from a recent systematic review on EUS-BD in the setting of double obstruction [57].

So far, literature describing GOO management with DS has been mostly published. However, given the abovementioned advantages of EUS-GE over DS, literature investigating newer procedural combination is awaited.

The first available study managing GOO with EUS-GE in the setting of double obstruction, is a retrospective study describing a series of 23 patients undergoing same session EUS-GE + EUS-HGS. Technical and clinical success were high, with 5 (21%) reported AEs (none severe or fatal) and 3 patients (14%) requiring reinterventions for recurrent or persistent jaundice [58].

A recent retrospective multicentric experience tried to compare different possible endoscopic combinations to treat patients with malignant double obstruction, focusing on recurrence-free survival^9^. The study included 103 procedure combinations and suggested that combinations using EUS-GE versus DS achieve better long-term outcomes and that either a transpapillary stent (via ERCP or antegrade stenting) or EUS-HGS are associated with decreased symptoms recurrence during follow-up, while EUS-CDS in this setting seems to be associated with reduced primary success and frequent dysfunction, independently from the fact that GOO has been solved by DS or EUS-GE[59].

As patients with concomitant obstruction and unresectable malignancies were historically treated by double-bypass surgery, a recent retrospective multicentric case–control study has suggested that same-session EUS-guided double bypass (EUS-GE + EUS-BD) achieves similar efficacy with reduced invasiveness than surgical double bypass (gastrojejunostomy + hepaticojejunostomy) [60]. This study reported a 94% clinical efficacy, an 11% rate of AEs (3.8% severe), no risk of dysfunction for EUS-GE, and a 7.5% of dysfunction for EUS-BD, suggesting improved outcomes of EUS-guided double bypass in more recent and high-volume experiences.

**CLINICAL QUESTION #13**

**In the setting of Duodenal stenosis, is there any difference between the extrahepatic and intrahepatic access for EUS-guided biliary drainage?**

**Statement #13**

**Si-EUS suggests that, in the specific setting of double obstruction, EUS-guided hepaticogastrostomy (EUS-HGS) may be favored over EUS-guided choledochoduodenostomy (EUS-CDS) due to longer stent patency.**

(*Level of evidence 4, Grade of recommendation: weak*).

**Summary of evidence**

The technical and clinical success rates of EUS-HGS and EUS-CDS are summarized in Supplementary Table 10**,** in which the results of an umbrella review, including three systematic reviews, are reported [61–63]. The umbrella review suggested that clinical and technical success and adverse events are similar between the two approaches. However, the populations of these systematic reviews also included patients without double obstruction, increasing the risk of bias. Some small multicenter studies [59, 64] showed that EUS-HGS may be favored over EUS-CDS due to longer stent patency.

**CLINICAL QUESTION #14**

**Does potential surgical resectability represent a contraindication to EUS-guided biliary drainage? Which biliary drainage should be performed in patients with resectable malignant distal biliary obstruction?**

**Statement #14**

**i-EUS suggests either ERCP or EUS-guided choledochoduodenostomy (EUS-CDS) as the first-line treatment of resectable MBDO.**

(*Level of evidence 4, Grade of recommendation: weak*).

**Summary of evidence**

The technical and clinical success rate of EUS-CDS with ECE-LAMS is similar to that of ERCP [5, 65–67]. EUS-guided drainage is associated with a lower rate of post-procedural and post-PD complications. The rate of post-procedural complications doubles if drainage follows a failed ERCP attempt, rather than being performed first [65]. A higher rate of overall complications and mild complications than EUS-guided drainage was described by Bang et al. In those cases, however, SEMS was used [5].

**CLINICAL QUESTION #15**

**How does EUS-BD compares with other endoscopic, surgical, or percutaneous approaches in achieving biliary drainage in patients with post-surgical anatomy?**

**Statement 15a**

**In patient with MBDO and surgical altered anatomy, treatment choice depends on disease extension and type of reconstruction. i-EUS suggests to consider EUS-BD over laparoscopic-/enteroscopy-assisted ERCP and PTBD in patient with Roux-en-Y anatomy.**

(*Level of evidence 4, Grade of recommendation: weak*).

**Statement 15b**

**i-EUS suggests to consider EUS-BD as a rescue strategy after failed ERCP with lateral-viewing or cap-assisted frontal-viewing endoscopes in patients with Billroth II reconstruction.**

(*Level of evidence 4, Grade of recommendation: weak*).

**Summary of evidence**

In patients with Billroth II reconstruction and malignant distal biliary stenosis, a lateral-viewed endoscope present similar clinical success rate to cap-assisted frontal-viewed endoscope with higher cannulation rate and lesser PEP rate [68–70]. After failed ERCP, EUS-BD (EUS-RV and EUS-anterograde biliary stenting) is preferable over percutaneous transhepatic biliary drainage (PTBD) due to better clinical success and lesser adverse events [71].

In patients with Billroth II or Roux-en-Y partial gastrectomy with distal malignant biliary obstruction in which papilla is not reached, EUS-HGS and or anterograde stent placement can be performed with a higher degree of clinical efficacy and shorter procedure time than enteroscopy-assisted ERCP. Whether or not this approach should be first-line therapy in this patient population is highly dependent on the indication for the procedure, the patient’s anatomy, and local practice and expertise [72–74].

In patients with RYGB, EUS-BD, especially Endoscopic Ultrasound-Directed transGastric ERCP (EDGE) procedure showed favorable technical and clinical success, comparable to laparoscopic ERCP, despite less invasive than the latter [75], and higher success rate than enteroscopy-assisted ERCP and PTBD [76]. Since there are no studies [77] regarding SAA and distal malignant obstruction, EDGE can be offered in expert center in Roux-en-Y bypass following multidisciplinary decision-making, according to ESGE guidelines. If EDGE procedure is not feasible, EUS-HGS can be considered. Choice of biliary drainage procedure in patients with SAA depends on surgical procedure performed, expertise, and equipment available at the center, interventional radiology, and surgical back up available.

**HOW TO TAKE CARE OF AND FOLLOW UP PATIENTS?**

**CLINICAL QUESTION #16**

**Who has to take care of patients requiring biliary drainage? Is multidisciplinary management associated with better outcomes?**

**Statement #16**

**I-EUS suggests multidisciplinary discussion of patients with distal malignant obstruction in whom the biliary drainage could impact on the main outcomes, in particular patients with resectable cancer, altered anatomy and double obstruction.**

(*Level of evidence 5, Grade of recommendation: weak*).

**Summary of evidence**

As concerns patients requiring biliary drainage, there are no data showing which clinician should be responsible for taking care of this specific condition and whether the management in a multidisciplinary team is associated with better outcomes. Moreover, these patients may be suffering from different pathologies, both benign and malignant. Some positive few data are available for oncological setting and especially for pancreatic cancer and cholangiocarcinoma. In this context, it has been proven that patients discussed at multidisciplinary meetings are more likely to receive more accurate diagnosis, staging, and more frequently appropriate treatment [78–81] (i.e., resectability, neoadjuvant and adjuvant chemotherapy), even if there is no evidence on the specific topic of biliary drainage. Furthermore, in case of malignant hilar biliary obstruction, multidisciplinary consultation to determine the most effective biliary drainage strategy is also recommended by ESGE [21]. Therefore, it seems reasonable to suggest a multidisciplinary discussion certainly when the biliary drainage could impact on the outcomes, as in the cases of resectable cancer, altered anatomy and double obstruction (biliary tree and duodenum). There are no defined criteria for a multidisciplinary team taking care of patients requiring biliary drainage.

**CLINICAL QUESTION #17**

**Does endoscopic biliary drainage need to be performed in a referral setting?**

**Statement #17**

**I-EUS suggests endoscopic biliary drainage to be performed in a setting where adequate competencies in interventional bilio-pancreatic management are available.**

(*Level of evidence 5, Grade of recommendation: weak*).

**Summary of evidence**

Several studies showed higher technical and clinical success rates and lower complication rates when EUS-guided biliary drainage is performed by experienced endoscopists.

A small study [82] including 31 patients showed significantly different procedure failure rate in the first three and last two years of experience (38 vs 11%, respectively). A Spanish survey [83] involving endoscopist with initial experience in biliary drainage showed lower technical success rate than previously reported in more experienced endoscopists. Moreover, a single-center study [84] on 101 patients reported a lower mortality rate among the first fifty patients during the first 5 years than in the last 51 patients during the last 2 years. The execution of at least the first twenty cases under a mentor’s supervision was suggested by Hara et al. [85]. A single operator study [86] aimed at defining the learning curve for EUS-BD showed that endoscopists experienced in EUS-BD are expected to achieve a reduction in procedural time as experience grows, with efficiency reached at 59 min and a learning rate of 32 cases. Another study [87] only focused on EUS-guided hepaticogastrostomy highlighted that the number of procedures to achieve technical proficiency was 24 for reducing and 33 to stabilize for procedural times. A very recent study [88] performed at EUS-BD beginner institutions reported that various experience-related factors (number of EUS-BD conducted by the institution and EUS screening, EUS-FNA, and EUS-guided drainage conducted by the operator, but not the operator’s experience with EUS-BD itself) may affect EUS-BD outcomes, pointing out the importance of the institution-conducted experience more than that of the single operator. However, also ESGE recommends therapeutic EUS procedures should be performed by endoscopists with adequate training and experience, at centers where interventional radiology and hepatopancreaticobiliary surgical expertise are available [21].

**CLINICAL QUESTION #18**

**Is a special diet suggested after biliary drainage?**

**Statement #18**

**i-EUS does not suggest any specific diet after EUS-guided biliary drainage to prevent stent dysfunction.**

(*Level of evidence 5, Grade of recommendation: weak*).

**Summary of evidence**

Stent dysfunction has been reported in published series up to 31%. Tumor over-in growth has been documented to be the primary cause of stent dysfunction; however, it is frequently described stent obstruction caused by food impaction, up to 40% [59, 89, 90]. Although food impaction has been reported in prospective and retrospective series as a cause of stent dysfunction, a special diet to prevent it has never been analyzed as a primary aim.

**CLINICAL QUESTION #19**

**Is there evidence to recommend the administration of antibiotics to reduce the risk of post-procedural complications after EUS-guided biliary drainage?**

**Statement #19**

**i-EUS does not suggest the routine use of antibiotic prophylaxis, to reduce the risk of post-procedural complications after EUS-guided biliary drainage.**

**i-EUS suggest that antibiotic prophylaxis should be offered in selected patients (e.g., immunocompromised patients, expected incomplete biliary drainage).**

(*Level of evidence 5, Grade of recommendation: weak*).

**Summary of evidence**

Infections may represent a fearful complication during EUS-guided procedures.

Despite this, the available literature does not show significant rates of infection after EUS-guided biliary drainage.

A large meta-analysis of one hundred fifty-five studies (7887 patients) showed that the pooled incidence of cholangitis after EUS-BD was 1.0% (95% CI 0.8–1.3), and the pooled incidence of major AEs (including but not limited to infections) was 0.6% (95% CI 0.3–0.9) [91].

According to another meta-analysis, the pooled rate of infection after EUS-BD was 3.8% (95% CI 2.8–5.1), without specifying, however, the rate of severe infections which are expected to be less prevalent [92].

Furthermore, there are currently no comparative studies demonstrating the efficacy of prophylaxis versus no antibiotic prophylaxis in preventing infections after EUS-BD.

However, some of these data can be translated from the setting of ERCP biliary drainage.

An old meta-analysis published in 1999 (5 RCTs, 1029 patients) found that the risk of sepsis or cholangitis following ERCP was not significantly decreased by routine antibiotic prophylaxis (OR 0.91, 95% CI 0.39 – 2.15) [93].

A recent meta-analysis of studies comparing the use of antibiotic vs non-antibiotic prophylaxis in patients undergoing elective ERCP (10 RCT, 1757 patients) confirmed the absence of significant difference in incidence of cholangitis [RD = − 0.02, 95% CI − 0.05, 0.02, *p* = 0.32], septicemia (RD = − 0.02, 95% CI − 0.06 to 0.01, *p* = 0.25), pancreatitis (RD = − 0.02, 95% CI − 0.06 to 0.01, *p* = 0.19), and all- cause mortality (RD = 0.00, 95% CI − 0.01 to 0.01, *p* = 0.71]. The antibiotic prophylaxis was associated with a 7% risk reduction in the incidence of bacteremia (RD = − 0.07, 95% CI − 0.14 to − 0.01, *p* = 0.03), but this has little clinical relevance [94].

Since post-procedural infections are most common in patients with expected incomplete drainage of biliary obstruction [95–97], current guidelines recommend antibiotic prophylaxis in patients with expected incomplete drainage of biliary obstruction [18, 98] [8, 9].

Similarly, patients with severe neutropenia and advanced hematologic malignancies are at increased risk for bacteremia and sepsis after GI endoscopy [99]. The protective effect of prophylactic antibiotics in these patients has not been studied. However, since this practice seems logical, guidelines recommend the use of antibiotic prophylaxis in immunocompromised patients with severe immunosuppression (absolute neutrophil count < 500 cells/mL, advanced hematologic malignancies, bone marrow transplantation, organ transplantation) [18, 98].

**CLINICAL QUESTION #20**

**Is there evidence to recommend the periprocedural administration of drugs to improve endoscopic outcomes after stent placement?**

**Statement #20**

**i-EUS does not suggest the administration of medical therapy to improve endoscopic outcomes after biliary drainage for malignant distal biliary obstructions.**

**iEUS does not suggest the use of ursodeoxycholic acid (UDCA) since it is not effective in preventing recurrent biliary obstruction after SEMS placement and may increase the risk of stent occlusion.**

(*Level of evidence 5, Grade of recommendation: *weak).

**Summary of evidence**

There are no studies in the literature investigating the potential role of drugs in improving endoscopic outcomes after LAMS placement, including stent patency. Regarding SEMS, recurrent biliary obstruction may occur primarily because of stent sludge occlusion, especially in fully covered SEMS with 7.9 to 11.1% occurrence rate and other factors [100–102].

A Cochrane meta-analysis (5 RCTs, 258 patients) was conducted to evaluate the effectiveness UDCA and/or antibiotics in prolonging stent patency and survival in patients with strictures of the biliary tract and endoscopically inserted stents [103]. Three trials, including 152 patients, investigated a combination of UDCA and antibiotics versus no treatment. The meta-analysis of these three trials does not show a significant treatment effect on the duration of stent patency (HR 0.58, 95% CI 0.22 to 1.54) or mortality (HR 0.99, 95% CI 0.68 to 1.43). Two trials with 106 patients compared antibiotics with no treatment. The pooled results of these two trials do not show significant effects of antibiotics on the duration of stent patency (HR 0.69 (95% CI 0.37 to 1.30)) or mortality (HR (fixed effect model) 1.23 (95% CI 0.72 to 2.08).

A recent propensity score–matched cohort analysis was conducted to assess the efficacy of ursodeoxycholic acid (UDCA) after SEMS placement for malignant distal biliary obstruction [104]. The study showed that the occlusion rate was 41.8% and 18.2% in the groups with and without UDCA, respectively (*p* = 0.0119). Median time to recurrent biliary obstruction was significantly longer in the control group than in the UDCA group (528 vs 154 days, *p* = 0.0381). Moreover, at multivariable analysis, UDCA was identified as the independent risk factor for reducing time to recurrent biliary obstruction (HR 2.28; 95% CI 1.06–4.88; *p* = 0.0348).

These data, therefore, do not allow to suggest the administration of medical therapy to improve endoscopic outcomes after biliary drainage for malignant distal biliary obstructions. The use of ursodeoxycholic to improve stent patency failed to demonstrate efficacy after SEMS placement, and its use is not recommended.

**CLINICAL QUESTION #21**

**Considering the interplay between ERCP and EUS, and the possibility of same-session procedures, should we obtain a “goal-based” informed consent for endoscopic biliary drainage, overcoming the concept of “technical-based” ones?**

**Statement #21**

**i-EUS suggests obtaining a “goal-based” informed consent for endoscopic malignant biliary drainage prior to either EUS-guided drainage or conventional ERCP.**

*(Level of evidence: 5; Grade of recommendation: weak)*

**Summary of evidence**

The synergy between EUS and ERCP and the potential for performing same-session EUS/ERCP procedures has raised implications that extend beyond the endoscopic room, particularly about obtaining proper informed consent (IC). Obtaining an effective IC is grounded in the fundamental human rights of autonomy and self-determination [105], and should encompass information regarding (i) the mechanisms of action, (ii) the balance between benefits and risks, and (iii) alternative treatment options.

Currently, various national and international endoscopy societies have introduced "technique-based" informed consent form templates for patients to sign before undergoing endoscopic procedures, aiming to establish a standardized approach. While these templates may be suitable for most purely diagnostic procedures, the field of interventional endoscopy has seen significant advancements in recent years, akin to developments in surgery. As a result, transitioning to a "goal-based" informed consent approach as happened in surgery may offer the most promising path to achieving our objectives within a single endoscopic session [106], thereby reducing the need for a second sedation, prolonged hospital stays, and associated costs.

## Discussion

MBDO is a key event in the natural history of pancreatic cancer and cholangiocarcinoma: it may deflect patient prognosis and affect the clinical management, such as access to surgery and oncological therapy [1–3]. A broad work of the current literature search was done to provide evidence on which experts participating to the consensus conference found a general agreement. On a total of 23 statements discussed, experts found an agreement above 80% in 21 of them, which were presented and discussed in the current manuscript.

Of the two rejected, one clinical question that was discussed regarded how biliary drainage should be performed in non-surgical candidate patients with distal MBO when ERCP fails. It was proposed that repeated ERCP or percutaneous rendezvous-ERCP should be considered as second-line choices after ERCP failure. That statement did not reach consensus (disagreement: 25.8%), with several opinions reported for being not in favor. The main one concerned the current role of EUS-BD, which showed efficacy and safety to be considered not inferior to ERCP as an upfront technique [8, 11], thus being certainly an alternative in case of ERCP failure. Other concerns regarded the definition of repeated ERCP (vs. second look after pre-cut) and the availability of EUS-BD in non-experienced centers.

The other clinical question that did not reach consensus regarded whether or not potential surgical resectability represents a contraindication to EUS-guided biliary drainage and which biliary drainage should be performed in patients with localized malignancies candidates to neoadjuvant therapy. It was proposed to state that either ERCP or EUS-BD could be as valid in the case of potentially resectable diseases scheduled for neoadjuvant chemotherapy. Evidence considered in support of that statement were that EUS-BD might be associated with a lower complication rate as compared to ERCP, thus reducing the length of hospital stay and earlier access to neoadjuvant treatment [65, 67]. The main reasons for not agreeing with the proposed statement (disagreement 21.2%) were that evidences about EUS-BD in the specific setting of the patient candidate to neoadjuvant therapy are still very limited and that therefore EUS-BD should be considered only in centers with broad experience.

While our statements are generally align with the ESGE guidelines recommendations [18, 42], our consensus offers a deeper insight into the management of MDBO. In particular, detailed guidance is provided on how to manage specific challenges in EUS-BD, the preferred techniques, and the type of stents to use. For example, our consensus includes recommendations on managing patients with concomitant biliary and gastric outlet obstructions, including the potential use of EUS-guided double bypass techniques. We also discuss using EUS-GBD as a rescue strategy when other EUS-BD approaches are not feasible, considerations for the prevention of stent dysfunction, and the use of prophylactic measures. Additionally, a detailed comparison of different types of stents (LAMS vs. SEMS) and their respective advantages, particularly for EUS-CDS, as well as specific recommendations on stent sizes and configurations, are included in the consensus.

While the ESGE guidelines recommend EUS-BD primarily as an alternative or rescue therapy after ERCP failure, we suggest EUS-CDS as a potential first-line treatment for unresectable MDBO, reflecting a shift toward considering EUS-BD earlier in the treatment algorithm. This suggestion is based on newly available evidence (Supplementary Table 1). We also make specific statements on the use of EUS-guided gastroenterostomy, suggesting this approach not only as an alternative to duodenal stenting, but as a primary therapeutic option in patients with life expectancy greater than 3 months in the setting of experienced centers.

This manuscript has limitations that mostly pertain the fact that the techniques have been entered the clinical practice only recently. Studies taken into account dealt with different patients’ features, obstruction localization, and technical approach. Hence, studies published so far lead to low-quality evidence of many recommendations. Such a context of evidence uncertainty is what lead us to provide our scientific and clinical community of an experts’ guidance that may support the readers in understanding what might be the most appropriate decisions.

Management of MBDO is experiencing many changes, in light of the several technical innovations that have entered the clinical practice. The main limitation of this work thus is that it pertains a novel field of research, with evidences that are still not mature or validated in different settings or populations. Still, and because of that, we believe that this work may serve as a guide for clinicians to better understand which options are nowadays available and to propose the adequate opportunities to patients they care.

## Supplementary Information

Below is the link to the electronic supplementary material.

Supplementary file1 (DOCX 22 KB)

Supplementary file2 (DOCX 36 KB)

Supplementary file3 (DOCX 42 KB)

Supplementary file4 (DOCX 86 KB)

Supplementary file5 (DOCX 23 KB)

## Abbreviations

AE: Adverse event
CBD: Common bile duct
CDS: Choledoco-duodenostomy
CPG: Clinical practice guideline
DPPS: Double pig-tail plastic Stents
DPS: Double pig-tail stent
ERCP: Endoscopic retrograde cholangiopancreatography
ES: Enteral stenting
EDGE: Endoscopic ultrasound-directed transgastric ERCP
ESGE: European society of gastrointestinal endoscopy
EUS: Endoscopic ultrasound
EUS-AG: EUS-guided antegrade
EUS-BD: EUS-guided biliary drainage
EUS-CDS: EUS-guided choledochoduodenostomy
EUS-GBD: EUS-guided gallbladder drainage
EUS-GE: EUS-guided gastroenterostomy
EUS-HGS: EUS-guided hepaticogastrostomy
EUS-RV: EUS-guided rendezvous
GOO: Gastric outlet obstruction
HGS: Hepaticogastrostomy
IC: Informed consent
i-EUS: Italian network of clinicians and scientists with a special interest in biliopancreatic endoscopy EUS
LAMS: Lumen-apposing metal stents
MDBO: Malignant distal biliary obstruction
OCEBM: Oxford centre for evidence-based medicine
PBD: Preoperative biliary drainage
PICO: Patient, intervention, comparison, outcome
PTBD: Percutaneous transhepatic biliary drainage
RCT: Randomized controlled trial
RYGB: Roux-en-Y gastric bypass
SAA: Surgically altered anatomy
SEMS: Self-expandable metal stents
UDCA: Ursodeoxycholic acid
