# Supplementary material for: Biliary drainage in patients with malignant distal biliary obstruction: results of an Italian consensus conference
Source: Surg Endosc. 2024 Sep 25;38(11):6207–26. doi: 10.1007/s00464-024-11245-4 (PMC11525304; doi:10.1007/s00464-024-11245-4)
Supplement: Supplementary file 1 — Supplementary file1 (DOCX 22 KB) [file 464_2024_11245_MOESM1_ESM.docx]

**SUPPLEMENTARY MATERIAL**

**i-EUS mission statement**

Interventional Endoscopy and Ultrasound (i-EUS) group is a nationwide community of interventional endoscopists with special interest in therapeutic EUS.

Over the past years, the development of new devices, techniques and instruments, along with the expanding indications in this filed, has brought to face new clinical and therapeutic scenarios, often of high technical and clinical complexity. Nowadays, it is of paramount importance to assess procedural outcomes, including clinical success, technical successes, and safety, analyzing data from large cohorts of patients and with long-term follow-up.

Gathering centers with different levels of expertise allows to perform a really constructive and agile comparison of various experiences and logistic models.

The i-EUS network therefore has the mission to share clinical experiences on this topic, to support and promote education initiatives in order to optimize the use of LAMS in clinical practice and to build clinical and scientific evidence on this topic.

The i-EUS group includes about 70 Centers all over Italy with different expertise in interventional EUS and LAMS use.

With this purpose, in the last 5 years, the i-EUS group was able to collect data and to publish more than 18 scientific papers (see below).

Among them there are also the results of the 1st i-EUS consensus on the management of pancreatic fluid collections (Capurso et al., Digestive and Liver Disease Jul 18:S1590-8658(24)00814-4). During the consensus meetings i-EUS has involved also surgeons, radiologist and clinicians.

***List of i-EUS publications:***

- Amato A et al., Gut 2022;71:1050–1052
- Binda C. et al., Gastrointestinal Endoscopy 2023 Nov;98(5):765-773
- Binda C. et al., Diagnostics 2024, 14, 413
- Binda C. et al., Medicina 2023, 59, 750
- Capurso et al., Digestive and Liver Disease Jul 18:S1590-8658(24)00814-4
- Coluccio C. et al., Digestive and Liver Disease 56 (2024) 159–169
- Fabbri C. et al., Endoscopy 2022; 54: 555–562
- Fabbri C. et al., Endoscopic Ultrasound 2022 Jan-Feb;11(1):59-67
- Facciorusso A. et al., Gastroointestinal Endoscopy 2022 Jun;95(6):1158-1172
- Facciorusso A. et al., Cancers 2022, 14, 3291
- Facciorusso A. et al., Digestive Endoscopy 2022; 34: 1459–1470
- Fugazza A. et al., Digestive and Liver Disease 54 (2022) 529–536
- Fugazza A. et al., Gastrointestinal Endoscopy 2022 May;95(5):896-904.e1.
- Fugazza A. et al., Gastrointestinal Endoscopy 2020 Mar;91(3):574-583
- Mauro A. et al., Medicina 2024,60,472
- Rizzo G. et al., Cancers 2023,15,5367
- Spadaccini M. et al., Medicina 2022, 58,331
- Tarantino I. et al., Medicina2022,58,532

**Choice of the experts for the Consensus Conference**

All the 70 centers of the i-EUS network were invited to participate to the work of the Consensus Conference. It is to be mentioned that i-EUS group includes members that belongs also to specialties other than endoscopy, such as clinical gastroenterologists, oncologists, radiologists, intrventional radiologists, surgeons, nutritionists.

A total of 61 experts from the i-EUS centers were able to attend the meeting and the following Delphi round. Besides the authors of the manuscript (who drafted the clinical questions, proposed the statements and performed literature search) the other contributors to the Consensus Conference and to the current paper are listed below.

**Contributors**

List of experts who contributed to the i-EUS consensus conference, together with the panel listed as authors of the manuscript.

| **Surname** | **Name** | **City** |
| --- | --- | --- |
| ARAGONA | GIOVANNI | PIACENZA |
| BADAS | ROBERTA | CAGLIARI |
| BALDUCCI | DANIELE | ANCONA |
| BARBERA | CARMELO | TERAMO |
| BERRETTI | DEBORA | UDINE |
| CAMELLINI | LORENZO | LA SPEZIA |
| CARROZZA | LUCIO | PALERMO |
| CENNAMO | VINCENZO | BOLOGNA |
| CUGIA | LUIGI | SASSARI |
| DEL VECCHIO BLANCO | GIOVANNA | ROMA |
| DE NUCCI | GERMANA | CARBAGNATE (MI) |
| DI MITRI | ROBERTO | PALERMO |
| DI PISA | MARTA | PALERMO |
| ERCOLANI | GIORGIO | FORLI-CESENA |
| FERRARA | FRANCESCO | PADOVA |
| GABBRIELLI | ARMANDO | VERONA |
| GIAMPALMA | EMANUELA | FORLI-CESENA |
| GIOVANNINI | MARC | MARSIGLIA |
| LARGHI | ALBERTO | ROMA |
| LI PETRI | SERGIO | PALERMO |
| MACCHIARELLI | RAFFAELE | SIENA |
| MANNO | MAURO | CARPI (MO) |
| MARCIANO | EMANUELE | PISA |
| MARIANI | ALBERTO | MILANO |
| MARUZZELLI | LUIGI | PALERMO |
| MUTIGNANI | MASSIMILIANO | MILANO |
| PAGANO | NICO | NOVARA |
| PAGANI | ANNA | PAVIA |
| PARODI | ANDREA | LAVAGNA (GE) |
| PIETRABISSA | ANDREA | PAVIA |
| PIRAS | ENRICO | CAGLIARI |
| POLLINO | VALERIA | CAGLIARI |
| RAIMONDO | DARIO | CEFALU' (PA) |
| STIGLIANO | SERENA | ROMA |
| TRAINA | MARIO | PALERMO |
| TRINGALI | ALBERTO | CONEGLIANO (TV) |
| TRINGALI | ANDREA | ROMA |
| VENEZIA | GIOVANNA | CUNEO |
| ZERBI | ALESSANDRO | ROZZANO (MI) |
