# Supplementary material for: Biliary drainage in patients with malignant distal biliary obstruction: results of an Italian consensus conference
Source: Surg Endosc. 2024 Sep 25;38(11):6207–26. doi: 10.1007/s00464-024-11245-4 (PMC11525304; doi:10.1007/s00464-024-11245-4)
Supplement: Supplementary file 2 — Supplementary file2 (DOCX 36 KB) [file 464_2024_11245_MOESM2_ESM.docx]

**Supplementary Table 1:** EUS-BD vs ERCP as first line approach

| **Author (Year)** | **Study Design** | **Patients (n)** | **Technical success** | | **Clinical success** | | **Adverse**  **Events** | | **Post-procedure pancreatitis** | |
| --- | --- | --- | --- | --- | --- | --- | --- | --- | --- | --- |
|  |  |  | EUS-BD | ERCP | EUS-BD | ERCP | EUS-BD | ERCP | EUS-BD | ERCP |
| **Paik (2018)** | RCT | 125 | 93.8% | 90.2% | 90% | 94,5% | 6.3% | 19.7% | 0% | 9% |
| **Bang (2018)** | RCT | 67 | 90,9% | 94,1% | 97% | 90% | 21,2% | 14,7% | 0% | 3% |
| **Park (2018)** | RCT | 28 | 92,8% | 100% | 100% | 92,8% | 0% | 0% | 0% | 0% |
| **Li (2019)** | MA | 220 | 92.8% | 92.7% | RR = 1.02; 95% CI: 0.93-1.11 | | RR = 1.64; 95% CI: 0.33-8.26 | |  | RR = 8.5; 95% CI: 1.03-69.91 |
| **Logiudice (2019)** | MA | 222 | 91.96% | 91.81% | 84.81% | 85.53% | RR = −0.06%; 95%CI: −0.23-0.12 | | - | - |
| **Kakked (2020)** | MA | 361 | 93.67% | 94.73% | 91.23% | 94.21% | 15.2% | 22.3% | 0% | 8% |
| **Jin (2020)** | MA | 302 | 92.8% | 92.7% | 90.5% | 91.5% | 15.3% | 27.9% | 0% | 11.5% |
| **Theo (2023)** | RCT | 156 | 96,2% | 76,3% | 93,7% | 90,8% | 16.5% | 17.5% | 0% | 2.6% |
| **Janet (2023)** | Prospective | 156 | 100% | 83.5% | 89,3% | 70.2% | 17,9% | 30,5% | 6,8% | 9.1% |

**Supplementary Table 2:** Meta-analyses comparing surgical outcomes with or without biliary drainage

| **Author, yr** | **Studies included** | **Population** | **Intervention** | **Outcomes** | **Remarks** | **Level of evidence** |
| --- | --- | --- | --- | --- | --- | --- |
| **Saleh, 2002** | 10 (2 RCTs, 8 retrospective) | 749 pts with benign or malignant distal biliary obstruction | Internal PBD vs. no PBD | OR for postoperative morbidity 0.79  (95%CI 0.36 – 1.73)  OR for postoperative mortality 0.81  (95%CI 0.33 – 1.99) |  | Moderate |
| **Sewnath, 2002** | 23 (5 RCTs, 18 retrospective) | 3155 pts with benign or malignant distal or proximal biliary obstruction | Internal/ external PBD vs. no PBD | OR for overall morbidity 1.99  (95%CI 1.25 – 3.16) and 1.64  (95%CI 1.20 – 2.26) in the RCTs and  retrospective studies, respectively  No significant difference in overall  mortality |  | Moderate |
| **Velanovich, 2009** | 15 (1 RCT, 14 retrospective) | Not reported | Internal/ external PBD  vs. no PBD | RD for postoperative morbidity  0.06% (95%CI –3.8% to 3.9%)  RD for overall mortality  –0.5% (95%CI –1.4% to 0.4%) |  | Low |
| **Qiu, 2011** | 14 retrospective | 1826 pts with  malignant obstructive  jaundice | Internal/ external PBD  vs. no PBD | No comparison of overall morbidity  OR for overall mortality 0.996  (95%CI 0.67 – 1.48) | No significant difference for any specific morbidity except postoperative  incision infection (OR 1.74, 95%CI 1.23 – 2.45) | Low |
| **Fang, 2012** | 6 RCTs | 520 pts with  benign or malignant  obstructive  jaundice | Internal (2 RCTs)/ external  (4 RCTs), PBD vs. no PBD | OR for overall serious morbidity 1.66 (95%CI 1.28 – 2.16)  OR for overall mortality 1.12 (95%CI 0.73 – 1.71) |  | Moderate |
| **Sun, 2014** | 14 (3 RCTs, 11 retrospective) | 2248 pts  with obstructive  jaundice | Intended internal PBD  vs. no PBD | OR for overall morbidity 1.11  (95%CI 0.76 – 1.64)  OR for overall mortality 0.74  (95%CI 0.52 – 1.05) | Subgroup analysis of 2 studies that used both plastic stents and SEMS for PBD vs. no PBD: NS for overall morbidity/ mortality (9 studies used plastic stents only and 3 studies did not discuss stent material) | Moderate |
| **Chen, 2015** | 44 (8 RCTs, 13 prospective,  23 retrospective) | 6286 pts with  malignant obstructive  jaundice | Internal/ external PBD vs. no PBD | OR of overall morbidity 0.99  (95%CI 0.81 – 1.22)  OR of overall mortality 1.03  (95%CI 0.76 – 1.38) | Subgroup analysis of RCTs: OR of morbidity 0.48 (95%CI 0.24 – 0.97) | Moderate |
| **Moole, 2016** | 26 (8 RCTs, 1 prospective, 17 retrospective | 3532 pts with  malignant obstructive  jaundice | Internal/ external PBD vs. no PBD | Pooled number of major morbidities  10.40 (95%CI 9.96 – 10.83) vs.  15.56 (95%CI 15.06 – 16.05)  OR of perioperative mortality 0.96 (95%CI 0.71 – 1.29) | Subgroup analysis: OR of major AEs for internal PBD vs. no PBD 0.48 (95%CI 0.32 – 0.74) | Moderate |
| **Scheufele, 2017** | 25 (3 RCTs, 22 retrospective) | 6214 pts with  malignant obstructive  jaundice | Internal/ external PBD vs. no PBD | OR of overall morbidity 1.40  (95%CI 1.14 – 1.72)  OR of perioperative mortality 0.91 (95%CI 0.66 – 1.26, P = 0.58) | - | Low |

**Supplementary Table 3a:** EUS-BD after failed ERCP, not comparative studies

| **Author, yr** | **Study type, centers** | **Studies included (tot)** | **N° Pts (tot)** | **Technical success (%)** | **Clinical success (%)** | **AE (%)** |
| --- | --- | --- | --- | --- | --- | --- |
| **Wang, 2016** | MA | 42 | 1192 | 95 | 92 | 23 |
| **Khan, 2016** | MA | 20 | 1186 | 94 | 90 | 17 |
| **Khashab, 2016** | P, multi | - | 96 | 96 | 90 | 11 |
| **Kunda, 2016** | R, multi | - | 57 | 98 | 95 | 7 |
| **Tsuchiya, 2018** | P, multi | - | 19 | 95 | 95 | 16 |
| **El Chafic, 2019** | R, multi | - | 67 | 96 | 100 | 8 (early) – 18 (late) |
| **Anderloni, 2019** | R, single | - | 46 | 93 | 98 | 11 |
| **Jacques, 2019** | R, multi | - | 52 | 89 | 100 | 4 (early) – 14 (late) |
| **Krishnamoorthi, 2020** | MA | 7 | 284 | 96 | 96 | 5 |
| **Venkatachalapathy, 2021** | P, multi | - | 20 | 100 | 95 (early) – 92 (late) | 10 |
| **On, 2021** | R, multi | - | 120 | 91 | 95 | 17 |
| **Tarantino, 2021** | P, single | - | 21 | 100 | 100 | 0 |
| **Vanella, 2022** | P, multi | - | 93 | 98 | 93 | 10 |
| **Fugazza, 2022** | R, multi | - | 256 | 93 | 96 | 10 |

**Supplementary Table 3b:** EUS-BD after failed ERCP, comparative studies

| **Author, yr** | **Study type, centers** | **Studies included (tot)** | **RCT included** | **N° Pts (tot)** | **Intervention (n)** | **Compared method (n)** | **Comment** |
| --- | --- | --- | --- | --- | --- | --- | --- |
| **Artifon, 2012** | RCT, single | - | - | 25 | PTBD (12) | EUS-CDS (13) | similar success, complication rate, cost, and quality of life |
| **Bapaye, 2013** | R, single | - | - | 50 | PTBD (26) | EUS-BD (25)  - EUS-CDS/HGS/AGS | EUS-BD superior in terms of success rates and complications |
| **Giovannini, 2015** | RCT , multi (abstract) | - | - | 41 | PTBD (21) | EUS-BD (20)  - EUS-CDS/HGS/AGS | Higher complication rate for PTBD |
| **Artifon, 2015** | RCT, single | - | - | 32 | surgical biliary bypass/hepaticojejunostomy (HJT) (16) | EUS-CDS (16) | Similar TS and CS, quality of life and survival. Procedural time less for EUS-CDS |
| **Khashab, 2015** | R, single | - | - | 73 | PTBD (51) | EUS-BD (22)  - EUS-CDS/RV | EUS-BD had decreased AE rate and significantly less costly due to the need for fewer reinterventions |
| **Lee, 2016** | RCT, multi | - | - | 66 | PTBD (32) | EUS-BD (34)  - EUS-CDS/HGS | EUS-BD fewer AE and lower reintervention |
| **Sharaiha, 2016** | R, single | - | - | 60 | PTBD (13) | EUS-BD (47)  - EUS-CDS/HGS | EUS-BD results in a lower need for reintervention, decreased rate of late AEs, and lower pain scores |
| **Sharaiha, 2017** | MA | 9 | 3 | 483 | EUS-BD | PTBD | EUS-BD higher CS, fewer AE, lower reintervention; more cost-effective |
| **Moole, 2017** | MA | 16 | 1 | 528 | EUS-BD | PTBD | EUS-BD higher successful biliary drainage rates and relatively fewer AE |
| **Sportes, 2017** | R, multi | - | - | 51 | PTBD (20) | EUS-HGS (31) | Similar TS, CS and AE. EUS.HGS showed lower overall reintervention rate and shorter length of hospital stay |
| **Miller, 2019** | MA | 3 | 3 | 132 | EUS-BD (77) | PTBD (55) | EUS-BD decreased risks of stent dysfunction |
| **Ginestet, 2021** | R, single | - | - | 95 | PTBD (45) | EUS-BD (50) | EUS-BD significantly better in CS, AE, hospital stay, costs |
| **Facciorusso, 2022** | MA | 5 | 5 | 217 | PTBD | EUS-CDS/EUS-HGS/ surgical hepaticojejunostomy | PTBD seems to have a slightly higher rate of adverse events |
| **Hayat, 2022** | MA | 10 | 6 | 1131 | EUS-BD (567) | PTBD (564) | Similar TS and CS, EUS-BD had lower acute and delayed AE |
| **Sawas, 2022** | R | - | - | 86 | PTBD (58) | EUS-CDS (28) | EUS‑CD showed a trend towards lower AEs and significantly lower need for reintervention. |

**Supplementary Table 3c:** Interval ERCP

| **First author**  **Year** | **Type of study** | **Patients, n** | **Inclusion criteria** | **Intervention** | **Malignancy included** | **Successful cannulation,**  **n (%)** |
| --- | --- | --- | --- | --- | --- | --- |
| **Ramirez, 1999** | R, single | 24 | Failed ERCP post NKS | Rpt ERCP by same endoscopist | Unclear | 21 (87.5%)  NKS - 4 (16.7%) |
| **Choudari, 2000** | P, single | 562 | Previous unsuccessful attempt |  | Unclear | 542 (96%) |
| **Kevans, 2010** | R, single | 19 | Failed ERCP post fistulotomy | Rpt ERCP by experienced endoscopist | Yes | 13 (68%) |
| **Kim, 2011** | R, single | 69 | Failed ERCP post NKS | Rpt ERCP in 3 days  -same endoscopist | Yes | 53 (76.8%)  NKS -7 (10.1 %) |
| **Donnellan, 2012** | R, single | 51 | Failed ERCP post needle knife |  | Unclear | 38 (74.5%) |
| **Pavlides, 2014** | R, single | 89 | Failed ERCP post pre-cut  sphincterotomy |  | Yes | 69/89 (77.5%)  3^rd^ attempt: 73/89 (82%) |
| **Colan-Hernandez, 2017** | R, 2 centres | 72 | Failed ERCP post pre-cut  sphincterotomy |  | Yes | 54 (75%) |
| **Penazola Ramirez, 2020** | P | 37 | Failed ERCP post pre-cut | Rpt ERCP 3 days | Yes | 29/37 (78.3%)  Pre-cut papillotomy: 1 (2.7%) |
| **Lo, 2021** | R, single |  | Failed ERCP post NKS | ERCP (31) ERCP-Rv (12) vs PTBD (25) vs Surgery (19) | Yes | 22/31(71%)  12/12 (100%) vs 25 (100%) vs 18 (94.7%),  p = 0.021 |
| **Flumignan, 2021** | R, single | 55 | Failed ERCP post NKS | Repeat ERCP 48 hours | Yes | 46 (83.6%) |
| **Deng, 2022** | R, single | 56 | Failed ERCP post NKS |  | Yes | 47 (83.9%)  NKS-15 (26.8%) |

**Supplementary Table 3d:** Percutaneous rendezvous ERCP

| **First author, Year** | **Type of study** | **Patients, n** | **Inclusion criteria** | **Malignancy included** | **Successful cannulation, (%)** |
| --- | --- | --- | --- | --- | --- |
| **Dowsett, 1989** | R | 72 | Benign and malignant indications | Yes | 83% |
| **Calvo, 2001** | R | 14 | choledocholithiasis | No | 93% |
| **Wayman, 2003** | R | 41 | PTD and PTE-rendezvous done in the same session vs different sessions | Unclear | All outcome measures better in the same session group |
| **Liu, 2010** | R | 27 | Repeated PE-RV in pts who received the same procedure previously and had recurrent obstructive jaundice | Unclear | 96% |
| **Tomizawa, 2014** | R | 23 | Single session | Yes | 88% |
| **Yang, 2017** | R | 42 |  | Yes | 93% |

**Supplementary Table 4:** EUS-BD and Sedation, not comparative studies

| **Author (Year)** | **Study Design** | **Patients (n)** | **Intervention** | **Type of sedation** | **Overall Technical Success** | **Overall Adverse Event (%)** | **Notes (type of stent -CDS)** |
| --- | --- | --- | --- | --- | --- | --- | --- |
| **Fugazza (2017)** | Retrospective | 256 | 256 CDS | Deep sedation or General Anesthesia | 93.3% | 10.5% | LAMS |
| **Minaga (2017)** | Retrospective | 30 | 30 HGS | Deep sedation | 96.7% | 33.3% | - |
| **Anderloni (2018)** | Retrospective | 46 | 46 CDS | Deep sedation | 93.5% | 11.6% | LAMS |
| **Nakai (2018)** | Prospective, nonrandomized | 34 | 34 CDS | Conscious sedation (moderate) | 97% | 15% | FCSEMS |
| **Bang (2018)** | Prospective, randomized | 67 | 33 CDS vs 34 ERCP | General Anesthesia | 90.9% CDS 94.1% ERCP | 21.2% CDS 14.7% ERCP | FCSEMS |
| **Kawakubo (2018)** | Retrospective | 82 | 26 CDS 56 ERCP | Conscious sedation | NA (clinical available) | 26.9% CDS 35.7% ERCP | FCSEMS |
| **Paik (2018)** | Prospective, randomized | 125 | 64 CDS vs 61 ERCP | Conscious sedation (moderate) | 93.8% CDS 90.2% ERCP | 6.3% CDS 19.7% ERCP | PCSEMS |
| **Park (2018)** | Prospective, randomized | 30 | 15 CDS vs 15 ERCP | Conscious sedation | 93% CDS 100% ERCP | 0% CDS 0% ERCP | FCSEMS |
| **Honjo (2018)** | Retrospective | 49 | 23 HGS CD 26 HGS MD | Conscious sedation | 97% CD 93.5% MD | 27.2% CD 16.1% MD | - |
| **Marx (2022)** | Prospective, randomized | 56 | 35 HGS 21 PTBD | General Anesthesia | 94.3% HGS 100% PTBD | - | - |
