# Supplementary material for: Biliary drainage in patients with malignant distal biliary obstruction: results of an Italian consensus conference
Source: Surg Endosc. 2024 Sep 25;38(11):6207–26. doi: 10.1007/s00464-024-11245-4 (PMC11525304; doi:10.1007/s00464-024-11245-4)
Supplement: Supplementary file 3 — Supplementary file3 (DOCX 42 KB) [file 464_2024_11245_MOESM3_ESM.docx]

**Supplementary Table 5a: Retrospective studies**

| **Study, year** | **Malignancies** | **Age** | **Gender, male** | **Primary outcome** | **Clinical success,%** | **Technical success,%** | **Long-term patency (months)** | **Adverse events (Aes), n (%)** | **Follow up (months)** |
| --- | --- | --- | --- | --- | --- | --- | --- | --- | --- |
| **Khashab, 2013** | Pancreatic cancer 18 Metastatic cancer 5 Duodenal cancer 4 Ampullary cancer 3 Cholangiocarcinoma 2 Gastric cancer 2 Liposarcoma 1 | 66.1 (± 11.6) | 18/35 (51.5%) | Efficacy andsafety | 32/33 (97%) | 33/35 (94%) | NA | EUS-RV vs EUS-BD 15.4% vs 10% | mean follow-up of 276 days |
| **Kawakubo, 2014** | Pancreatic cancer, n (%) 42 (66) Bile duct cancer, n (%) 5 (8) Gallbladder cancer, n (%) 1 (2) Ampullary cancer, n (%) 9 (14) Metastatic lymph nodes, n (%) 7 (10) | Median (IQR)  Overall 72 (66–79) | 35 (55) | Technical success | NA | Overall 95% 61/64 | 3-month dysfunction-free patency rate were 21% and  80% for EUS-CDS and 32% and 51% for EUS-HGS | 12/64 (19%) | Median 94 ( range 9–1593) |
| **Khashab, 2015** | pancreatic adenocarcinoma (n = 43), Cholangiocarcinoma (n = 12), metastatic carcinoma (n = 12) Ampullary cancer (n= 3),Duodenal cancer (n = 1), Gastric cancer (n= 1), Liposarcoma(n = 1). | EUS-BD 64.9 ± 12.5  PTBD 66.9 ± 12.5 | EUS-BD 12 (54.5)  PTBD 29 (56.9) | NA | EUS-BD 86.4%,  PTBD 92.2% | 19/22 86.4% EUS-BD  51/51 100% PTBD | EUS-BD 198 days,range 4–986 PTBD 184 days, range 1–1,414 | 4/22 (18.2%) EUS-BD  20/51 (39.2%)PTBD | 320 days (excluded death for disease progression) |
| **Imai, 2016** | Pancreatic cancer, followed by lymph node metastasis, bile duct cancer, and malignant lymphoma. | 67.3 ± 13.9 | 8/12, (66%) | Technical and clinical success rate, AEs rate, survival, stent dysfunction | 91.7%, (11/12) | 100% (12/12) | NA | 2/12 (16.6%) | NA |
| **Kunda, 2016** | Pancreatic, 38 (66.6 %) Ampullary, 7 (12.3 %) Duodenal, 6 (10.5 %) Breast in 2 (3.5 %) Stomach, 2 (3.5 %) Colon, 1 (1.8 %) Uterine, 1 (1.8 %) | Median 73 years (range 49–93) | Male/female, n, 31/26 | safety, technical and clinical success | 54/56 (96.4%) | 98.2% (56/57) | NA | 7% (4/57) | Mean 151 ± 145 days (15 alive at the end of FU) |
| **Khashab, 2016** | pancreatic cancer 65 [54 %] | EUS-CDS 67.6 ± 13  EUS-HGS 63.6 ± 13.8 | EUS-CDS 32/60 (53.3%)  EUS-HGS 38/61 (62.3%) | Technical success and safety | EUS-CDS 85.5%  EUS-HGS 82.1% | 112/121 (92.56 %)  EUS-CDS93.3 %  EUS-HG 91.8 % | The 1-year stent patency  EUS-CDS group 98% vs HGS 60% | EUS-HGS 12/61 19.67%  EUS-CDS 8/60 13.3 %, | Mean follow-up 151±159 days |
| **Rai, 2018** | Periampullary carcinoma 15 (50%) Gallbladder carcinoma 6 (20 %) Carcinoma head of pancreas 6 (20%) Distal cholangiocarcinoma 3 (10 %) | Median 60.5 (34 – 80) | 16 (53.3%) | technical success, clinical success, stent patency rate and occurrence of adverse events | 28/30 93% | 28/30 (93%) | 3-month dysfunction-free stent patency rate was 83% | 3/30 (10% | 12 weeks |
| **Jacques, 2019** | Pancreatic adenocarcinoma 43 (82.7) Cholangiocarcinoma 2 (3.8) Degenerated IPMN 2 (3.8) Duodenal lymphoma 1 (1.9) Peritoneal carcinomatosis 1 (1.9) Duodenal carcinoma 2 (3.8) Stones 1 (1.9) | Mean 78 ( range 61 – 92) | 25 male, 48% | Technical and clinical success | 100% 46/46 | 46/52 (88.5%) | NA | 2/52 (3.8%) | Mean 157 days |
| **Anderloni, 2019** | Pancreatic cancer 40 (87%) Duodenal cancer 3 (6.5%) Ampullary cancer 2 (4.3%) Distal cholangiocarcinoma 1 (2.2%) | median age, 73.1 ± 12.6 years | 24/46 (52.2%) | technical and clinical success | 42/43 (97.7%) | 43/46 (93.5%) | The 3-month and 6-month stent patency rates were 87% and 70% | 5/46 (11.6%) | mean follow-up was 114.37 days (95% CI,73.2-155.4) |
| **El Chafic, 2019** | Peri-ampullary cancer 56 (83.6%) Metastatic cancer 11 (16.4%) | mean age  68.8 ± 11.8 years | 37/67 (55.2%) | Technical success | 40 (100%) = only in pts followed for more than 4 weeks | 64/67 (95.5%) | NA | 5/67 (7.5%) | median follow-up 17.0 weeks; range 4–81 |
| **Jacques, 2020** | Pancreatic cancer 54 (77%) Cholangiocarcinoma 3 (4%) Ampulloma 4 (6%) Duodenal carcinoma 2 (3%) Other 7 (10%) | Mean 75 years (range: 61–92 years | 38 male, 54.3% | Technical success rate. | 98.6% (69/70) | 98.6% (69/70) | the 6-month stent  patency rate was 91.4% | 9/70 12.9% | Median 153 days |
| **On, 2021** | Pancreatic ductal adenocarcinoma 77 (64.2%) Duodenal adenocarcinoma 14 (11.7%) Ampullary adenocarcinoma 9 (7.5%) Distal cholangiocarcinoma 8 (6.7%) Metastatic disease from other primaries 12 (10%) | Median 73 years (range 43-94) | 66/120 (55%) | Technical and clinical success | 92/97 (94.8%)  97 patients had data for Clinical success | 109/120 (90.8%) | 91.7% patent (100/109) | 17.5% (n = 21) | Median follow-up period in 117 patients was 70 days |
| **Issa, 2021** | Undefined unresectable malignant distal bile duct obstruction | mean age was  68 years (standard deviation [SD] 13 years | 15/28 (54%) | Efficacy and safety | 26/28 (93%) | 100% (28/28) | Stent patency at > 30 days was 82%. | 5/28 (17.9%) | median of 33 months (range 3 – 64) |
| **Tarantino, 2021** | 66.6% (n = 14) pancreatic tumor 14.2% ampullary tumor, 14.2% n = (3) dital cholangiocarcinoma 4.7% (n = 1) undetermined tumor | NA | 42.9% (n = 9) | clinical success | 100% 21/21 | 100% 21/21 | NA | 0% 0/21 | Median follow-up 188 days (range 8-554) |
| **Ishiwatari, 2022** | Pancreas 54 Biliary 8 Others 20 | Median 71 (IQR 64-78) HGS  Median 72 (IQR 64-76) HGAS | HGS 33 (56.9)  HGAS 23 (60.5) | Time to recurrent biliary obstruction, TRBO (234 days vs not reached) | 55 (94.8) for EUS HGS  37 (97.3) for EUS-HGAS | NA | 15 RBO for HGS and 5 RBO for HGAS | 8 (13.8) for HGS  7 (18.4) for HGAS | NA |
| **Di Mitri, 2022** | Pancreatic cancer (30/36 – 83.3%), duodenal carcinoma (1/36 – 2.8%), cholangiocarcinoma (3/36 – 8.3%), ampullary neoplasia (2/36 – 5.6%) | Median 75 (IQR 61–82.5) | 18/36 (50%) | Technical and clinical success | 100% 36/36 | 29/36 (80.6 %) | NA | 9/36 (25%) | Median 160 (102–205) Days |
| **Ginestet, 2022** | Pancreatic cancer 42 (85.7%) Diastal cholangiocarcinoma 2 (4%) Ampullary carcinoma 3 (6%) Others 2 (4%) | EUS-BD Mean 76.5  PTBD mean 74.3 | 30/50 (60%) | Clinical success rates at 1 month | 45/50 (89.3%) | 49/50 (97.9%) | 46/50 (92%) during FU | 2/50 (4%) | NA |
| **Fugazza, 2022** | Pancreatic adenocarcinoma (75%), ampullary cancer (8.6%) , cholangiocarcinoma (6.6%) | mean age of 73.9 ± 12.6 | 55.1% (141/256) | Technical and clinical success | 230/239 (96.2%) | 239 of 256 (93.3%) | NA | 25/239 (10.5%) | mean follow-up was 151 ± 162 days |

**Supplementary Table 5b: RCTs**

| **Study, year** | **Malignancies** | **Age** | **Gender, male** | **Primary outcome** | **Clinical success,%** | **Technical success,%** | **Long-term patency (months)** | **Adverse events (Aes), n (%)** | **Follow up (months)** |
| --- | --- | --- | --- | --- | --- | --- | --- | --- | --- |
| **Artifon, 2012** | Pancreatic adenocarcinoma 16, Ampullary adenocarcinoma 1, Hematologic neoplasia 2, Cholangiocarcinoma 2, Metastasis 3, Gastric carcinoma 1. | EUS-CD 63.4 ± 11.1  PTBD 71 ± 11.9 | EUS-CD 9 (69.2%)  PTBD 8 (66.6%) | Clinical success | 100% | 100% | NA | EUS-CD (2/ 13; 15.3%)  PTBD: 3 complications (3/12; 25%) | 79.6 days in the EUS-CDS group  74.9 days in the PTBD group |
| **Artifon, 2015** | Pancreatic adenocarcinoma (n=33), Metastatic adenopathy (n=8), Ampullary carcinoma (n=4), Neuroendocrine tumor (n=2), Gallbladder cancer (n=1), Duodenal carcinoma (n=1) | EUS-CDS 65 ± 12.2  EUS-HGS 68.1 ± 19.5 | EUS-CD 11 (50%)  EUS-HPG 11 (45.8%) | Technical and Clinical success | 91% for HPG  77% for CDS | 96% for HPG 91% for CDS | NA | Overall 16.3%  20% for the HPG 12.5% for the CDS | 90 days |
| **Lee, 2016** | Cholangiocarcinoma 21, Pancreatic adenocarcinoma 24, Gallbladder carcinoma 8, Ampullary adenocarcinoma 1, Metastasis 4, Gastric carcinoma cancer 5, Duodenal carcinoma 3 | EUS-BD 66.5 (40–83)  PTBD 68.4 (52–82) | EUS-HG NR  PTBD 24 (75%) | Technical success | 28/32, 87.5% (EUS-BDS)  27/31, 87.1% (PTBD) | 32/34, 94.1% (EUS-BDS)  31/32, 96.9 (PTBD) | 228 d EUS-BDS  220 d PTBD | 3/32, 8.8% (EUS-BDS)  17/31, 54.8% (PTBD) | 3 months |
| **Minaga, 2019** | Pancreatobiliary cancer (n=41) Other (n=6) | EUS-CD 73 (41–83)  EUS-HGS 72.5 (46–88) | EUS-CD 10 (43.4%)  EUS-HG 14 (58.3%) | Technical success | 100% HGS  95.7% CDS | 87% HGS  82.6% CDS | 306 Days median for HGS | 28.6% HGS and CDS 21.1% | NA |

**Supplementary Table Andrea 5c. Prospective studies**

| **Study, year** | **Malignancies** | **Type of BD** | **Age** | **Gender, male** | **Primary outcome** | **Clinical success,%** | **Technical success,%** | **Long-term patency (months)** | **Adverse events (AEs), n (%)** | **Follow up (months)** |
| --- | --- | --- | --- | --- | --- | --- | --- | --- | --- | --- |
| **Hara, 2011** | Unresectable malignant tumor | EUS-CDS | Mean 67.9 ± 10.9 | 7/18 men (38.9%) | Safety | 100 % (17 / 17) | 94 % (17 / 18) | 9/18 Median stent patency was 272 days | 3/18 (17 % ) | median: 163 days; range: 46 – 484 days |
| **Song, 2012** | Pancreatic cancer (n = 9), ampulla of Vater cancer (n = 2), renal cell carcinoma (n = 1), Advanced gastric cancer (n = 1), lymphoma (n = 1), duodenal cancer (n = 1). | EUS-CDS | 61 (range: 30-91 years) | 8, (53%) | NA | 100% among the 13 technically successful | 86.7% (13/15) | Mean patency 264 days | 4/13, 30.8% | median: 186 days, range: 52-388 days |
| **Cho*, 2017** | Pancreatic cancer (n = 25), cholangiocarcinoma (n = 12), gallbladder cancer (n = 4), metastatic cancer (n = 5; one case each of cervix cancer, ovarian cancer, advanced gastric cancer and 2 cases of colon cancer), hepatocellular carcinoma (n = 2), and pancreatic neuroendocrine tumor (n = 2). | EUS-CDS | median age 65.5 years (range: 29–86 years) | 29/54 (53.7%) | NA | Overall 94.4% (51/54) | 100% (54/54) | Mean stent patency 166.3 days and 329.1 days in the EUS-HGS and EUS-CDS groups | 9/54 (16.6%) | median: 148.5, IQR: 79.7-244 days |
| **Tsuchiya, 2018** | Pancreatic cancers (n = 10), cholangiocarcinomas (n = 2), metastatic renal cancers (n = 2), ampullary cancer (n = 1), metastatic colon cancer (n = 1), sarcoma (n = 1), duodenal cancer (n = 1), and metastatic gastric cancer (n = 1) | EUS-CDS | 70.6 ± 13.9 years | 12/19 male | NA | 18/19 (94,74%) | 19/19 (100%) | 73.7% (14/19) during median FU time | Overall AEs 36.8% (7/19) | median, 145 days; range, 12-819 [mean 205 ± 187.9] |
| **Itonaga, 2019** | Pancreatic cancer 11 (55%) Distal bile duct cancer 5 (25%) Ampullary cancer 3 (15%) Lymph node metastasis of colon cancer 1 (5%) | EUS-CDS | Mean 70.7 (33 90) | 12 (60%) | Clinical efficacy and safety | 19/19 100% | 19/20 95% | 15/19 (78.9%) patent at 97 days | 1/20 (5%) | Mean 132 ± 94 days |
| **Ragab, 2023** | Advanced pancreatic/ ampullary tumor with no duodenal obstruction 58 (63.7%) Advanced pancreatic/ ampullary tumor with duodenal obstruction 17 (18.7%) Altered anatomy (with tumor recurrence post-whipple operation) 7 (7.7%) Cholangiocarcinoma (Mid and Distal CBD) 5 (5.5%) Undifferntiated CBD malignancy 4 (4.4%) | EUS-CDS | median age: 61 years, (IQR 55-69) | 59/91 (64.8%) | Technical and clinical success | 80/85 (94.1 %) | 85/91 (93.4%) | NA | 10/85 (11,8%) | NA |

*In this study EUS-HGS was performed for proximal biliary obstruction, while EUS-CDS for mid-to-distal biliary obstruction

**Supplementary Table 6**

| **Author, year** | **Design** | **Indication** | **Stent** | **Patients number** | **Tech. Success rate** | **Clin. Success rate** | **AE**  **Rate** | **Reintirvention**  **Rate** |
| --- | --- | --- | --- | --- | --- | --- | --- | --- |
| **SEMS** |  |  |  |  |  |  |  |  |
| Fabbri, 2011 | Retrospective | Failed ERCP | SEMS | 9 | 100% | 100% | 11.1% | 0% |
| Park, 2011 | Prospective | Failed ERCP | SEMS | 26 | 92.3% | 84.6% | 19.2% | 0% |
| Song, 2012 | Prospective | Failed ERCP | SEMS | 15 | 86.7% | 86.7% | 46.7% | 26.7% |
| Hara, 2012 | Prospective | Primary | SEMS | 18 | 94.4% | 88.9% | 11.1% | NR |
| Kim, 2012 | Retrospective | Failed ERCP | SEMS | 9 | 100% | 100% | 33.3% | 22.2% |
| Artifon, 2012 | RCT | Failed ERCP | SEMS | 13 | 100% | 100% | 15.4% | NR |
| Khashab, 2013 | Retrospective | Failed ERCP | SEMS | 15 | 100% | 93.3% | 20.0% | 6.7% |
| Poincloux, 2015 | Retrospective | Failed ERCP | SEMS | 26 | 100% | 100% | 7.7% | NR |
| Artifon, 2015 | RCT | Failed ERCP | SEMS | 16 | 87.5% | 62.5% | 18.8% | 18.8% |
| Amano’ 2016 | Prospective | Failed ERCP | SEMS | 11 | 100% | 100% | 18.2% | 0% |
| Khashab, 2016 | Prospective | Failed ERCP | SEMS | 56 | 96.4% | 96.4% | 7.1% | NR |
| Guo, 2016 | Retrospective | Failed ERCP | SEMS | 14 | 100% | 100% | 7.1% | 7.1% |
| Ogura, 2016 | Retrospective | Failed ERCP | SEMS | 13 | 100% | 100% | 46.2% | 46.2% |
| Lee, 2016 | RCT | Failed ERCP | SEMS | 8 | 87.5% | 87.5% | NR | NR |
| Lu, 2017 | Retrospective | Failed ERCP | SEMS | 17 | 100% | 100% | 23.5% | 11.8% |
| Cho, 2017 | Prospective | Failed ERCP | SEMS | 33 | 100% | 100% | 30.3% | 12.1% |
| Huang, 2017 | Prospective | Failed ERCP | SEMS | 12 | 91.7% | 91.7% | 0% | NR |
| Rai, 2017 | Retrospective | Failed ERCP | SEMS | 30 | 93.3% | 93.3% | 10.0% | NR |
| Park, 2018 | RCT | Primary | SEMS | 14 | 92.9% | 92.9% | 14.2% | 0% |
| Alvarez-Sanchez, 2018 | Retrospective | Failed ERCP | SEMS | 12 | 100% | NR | 16.7% | 8.3% |
| Bang, 2018 | RCT | Primary | SEMS | 33 | 90.9% | 96.9% | 21.2% | 3.0% |
| Paik, 2018 | RCT | Primary | SEMS | 32 | 90.6% | 87.5% | 15.6% | 18.7% |
| Nakai, 2018 | Prospective | Primary | SEMS | 34 | 97.1% | 97.1% | 14.7% | 11.7% |
| Itonaga, 2018 | Prospective | Failed ERCP | SEMS | 20 | 95.0% | 95.0% | 5.0% | 20.0% |
| Minaga, 2019 | RCT | Failed ERCP | SEMS | 23 | 82.6% | 95.7% | 17.4% | 4.3% |
| Koga, 2021 | Retrospective | Primary | SEMS | 41 | 97.6% | 87.8% | 19.5% | 56.1% |
| de Benito Sanz, 2022 | Retrospective | Failed ERCP | SEMS | 20 | 100% | 95.0% | 20.0% | 10.0% |
|  |  |  |  |  |  |  |  |  |
| **Authors** | **Design** | **Indication** | **Stent** | **EUS-CDS** | **TSR** | **CSR** | **AER** | **RR** |
| **LAMS** |  |  |  |  |  |  |  |  |
| Kunda, 2016 | Retrospective | Failed ERCP | LAMS | 57 | 98.2% | 94.7% | 15.8% | 8.7% |
| Tsuchiya, 2017 | Prospective | Failed ERCP | LAMS | 19 | 100% | 94.7% | 36.8% | 21.1% |
| Jacques, 2018 | Retrospective | Failed ERCP | LAMS | 52 | 88.5% | 88.5% | 17.3% | 15.3% |
| Anderloni, 2019 | Retrospective | Failed ERCP | LAMS | 46 | 93.5% | 91.3% | 10.9% | 8.7% |
| El Chafic, 2019 | Retrospective | Failed ERCP | LAMS | 67 | 95.5% | 100% | 17.9% | 10.4% |
| Jacques, 2020 | Retrospective | Failed ERCP | LAMS | 70 | 98.6% | 98.6% | 12.9% | 5.7% |
| Mangiavillano, 2021 | Retrospective | Failed ERCP | LAMS | 7 | 100% | 100% | 14.3% | NR |
| Di Mitri, 2022 | Retrospective | Failed ERCP | LAMS | 31 | 80.6% | 100% | 100% | NR |
| Fugazza, 2022 | Retrospective | Failed ERCP | LAMS | 256 | 93.3% | 96.2% | 10.5% | 9.2% |
| Manta, 2022 | Retrospective | Failed ERCP | LAMS | 15 | 100% | 93.3% | 6.7% | 13.3% |
| Ginestet, 2022 | Retrospective | Failed ERCP | LAMS | 50 | 100% | 90% | 2.0% | NR |
| de Benito Sanz, 2022 | Retrospective | Failed ERCP | LAMS | 37 | 100% | 100% | 13.5% | 5.4% |
| Teoh, 2023 | RCT | Primary | LAMS | 79 | 96.2% | 93.7% | 16.5% | 8.9% |

**Supplementary Table 7**

|  | **Estimates** [95% confidence interval] | **Inconsistency** | **Publication bias** |
| --- | --- | --- | --- |
| *Overall* |  |  | *Egger’s test* |
| Technical success rate (38 studies; 1277 patients) | 95.6% [94.1 – 96.9] | I^2^ 25.5% | 0.81 |
| Clinical success rate (37 studies, 1265 patients) | 93.9% [91.8 – 95.7] | I^2^ 45.2% | 0.57 |
| Adverse event rate (37 studies, 1269 patients) | 15.6% [12.7 – 18.8] | I^2^ 49.6% | 0.02 |
| Reintervention rate (28 studies, 1026 patients) | 12.7% [9.1 – 16.7] | I^2^ 67.1% | 0.35 |
| *Self-expandable metal stents* |  |  |  |
| Technical success rate (27 studies; 570 patients) | 94.7% [92.8 – 96.4] | I^2^ 0.0% | 0.95 |
| Clinical success rate (26 studies, 558 patients) | 93.2% [90.5 – 95.5] | I^2^ 29.9% | 0.81 |
| Adverse event rate (26 studies, 562 patients) | 17.9% [14.3 – 21.8] | I^2^ 28.2% | 0.22 |
| Reintervention rate (20 studies, 407 patients) | 13.7% [7.9 – 20.8] | I^2^ 73.6% | 0.33 |
| *Lumen-apposing metal stents* |  |  |  |
| Technical success rate (12 studies; 707 patients) | 96.7% [94.2 – 98.5] | I^2^ 55.1% | 0.24 |
| Clinical success rate (12 studies, 707 patients) | 94.8% [91.3 – 97.5] | I^2^ 65.3% | 0.26 |
| Adverse event rate (12 studies, 707 patients) | 12.3% [8.2 – 17.0] | I^2^ 62.9% | 0.58 |
| Reintervention rate (9 studies, 619 patients) | 9.7% [7.5 – 12.2] | I^2^ 0.0% | 0.16 |
