# Supplementary material for: Biliary drainage in patients with malignant distal biliary obstruction: results of an Italian consensus conference
Source: Surg Endosc. 2024 Sep 25;38(11):6207–26. doi: 10.1007/s00464-024-11245-4 (PMC11525304; doi:10.1007/s00464-024-11245-4)
Supplement: Supplementary file 4 — Supplementary file4 (DOCX 86 KB) [file 464_2024_11245_MOESM4_ESM.docx]

**Supplementary Table 8**

| **Author, year** | **Design** | **Studies included** | **RCTs included** | **EUS-GE**  **(N° pts)** | **SEMS**  **(N° pts)** | **Surgical GE**  **(N° pts)** | **Technical**  **success**  **(EUS-GE vs SEMS)** | **Clinical**  **Success**  **(EUS-GE vs SEMS)** | **GGO recurrence**  **(EUS-GE vs SEMS)** | **Overall adverse events** | **Conclusions** |
| --- | --- | --- | --- | --- | --- | --- | --- | --- | --- | --- | --- |
| Chen , 2017 | Retrospective | - | - | 30 | 52 | - | 86.7 vs 94.2% (p=0.2) | 83.3 vs 67.3% (p=0.12) | 4.3 vs 28.6% (p=0.015) | 16.7 vs 11.5 (p=0.5) | - EUS-GE as effective as ES - Minimal invasive - Comparable AEs |
| Ge, 2019 | Retrospective | - | - | 22 | 78 | - | 100% both groups | 95.8 vs 76.3%, (p=0.042) | 8.3 vs 32% (p=0.021) | 20.8 vs 40.2 p=0.098 | - EUS-GE: minimally invasive alternative for selected pts - Centers with extensive therapeutic EUS experience. |
| Boghossian MB, 2021 | Metanalysis | 7 | 0 | 513 (total) | | | 93.3% vs 98.3%  (p=0.68) | 88.3% vs 78.%  (p=0.01) | 6.7 vs 28.6  (p<0.01) | 11.66 vs 31.32  (p=0.002) | - EUS-GE: Higher clinical success - EUS-GE: fewer severe Aes - EUS-GE: decreased stent obstruction and need for reintervention |
| Van Wanrooij, 2022 | Retrospective | - | - | 88 | 88 | - | 94 vs 98 % (p=0.44) | 91 vs. 75% (p = 0.008) | 1 vs. 26% (p<0.001) | 10 vs 21% (p=0.09) | - EUS-GE: higher clinical success - EUS-GE: lower stent dysfunction - Similar safety   *EUS-GE may be preferred*  *over duodenal stenting in patients with malignant GOO.* |
| Sánchez-Aldehuelo, 2022 | Retrospective | - | - | 79 | 97 | - | 93.7 Vs. 92.8 %  (p=0.82) | 92.4 vs. 83.5 % (p=0.08) | 8.8 vs 25.8% (p=0.004) | 10.1 vs 10.3% (p=0.74) | - EUS-GE may improve long-term patency - similar safety profile. |
| Krishnamoorthi R, 2022 | Metanalysis | 61 | 0 | 5772 (total) | | | EUS-GE:95.3%  SEMS:99.4%  Surgical GJ: 99.9%  (p=0.0048) | EUS-GE:88.9%  SEMS: 89.0%  Surgical GJ: 92.3%  (p=0.49) | EUS GE: 4%  SEMS: 28.7  Surgical GJ: 16.9% | EUS-GE: 21.9%  SEMS:18.7%  Surgical GJ: 23.8%  (p=0.32) | EUS-GE vs DS vs SGJ: similar clinical success and AEs rate  EUS-GE: lowest technical success rate, lowest GOO recurrence  DS: Highest stent occlusion rate |
| Vanella, 2023 | Prospective cohort study | - | - | 28 | 28 | - | 94.6 Vs. 100 %  (p=0.32) | 100 vs 75% (**p=0.006**) | 3.7 vs. 33.3% (**p=0.007**) | 7.1 vs 25%  (p=0.07) | - EUS-GE: excellent efficacy - EUS-GE: acceptable safety - EUS-GE: long-term patency,   *EUS-GE as first line strategy for mGOO, where adequate expertise is*  *available.* |

**Supplementary Table 9**

| **Duodenal stent + ERCP** | | | |
| --- | --- | --- | --- |
| **Study** | **Design** | **Patients** | **Results** |
| Mutignani M. *2007* | Retrospective, Single Center | 64 | ERCP + Duodenal Stent (in some cases, ERCP performed before the onset of duodenal obstruction):  ERCP success: 90% (10% required PTBD-rendez-vous)  AEs: 6% early; 16% late |
| Khashab M. *2014* | Retrospective, Multicenter | 38 | ERCP in patients with DS in place:  ERCP success: 34%  Rescue strategies were EUS-BD (59%) and PTBD (41%) |
| Staub J, 2018 | Retrospective, Multicenter | 71 | ERCP in patients with DS in place:  ERCP success: 85% (higher in type 3 stenoses, followed by type 1 and, lastly type 2 stenoses)  AEs: 5% |
| Simoes PK, 2022 | Retrospective, Single Center | 64 | ERCP in patients with DS in place:  ERCP success: 59% (higher in type 1 and 3 stenoses versus type 2: 83% and 92% vs. 42%, p<0.01)  AEs: 11% |
| **Duodenal stent + EUS-BD** | | | |
| **Study** | **Design** | **Patients** | **Results** |
| Ogura T, 2014 | Retrospective, Single Center | 39 | EUS-BD + DS  Comparison of EUS-HGS versus EUS-CDS:  Significantly longer biliary patency: median 133 vs. 37 days; p=0.045.  On logistic regression analysis EUS-CDS was associated with increased AEs, particularly reflux cholangitis |
| Hamada T, 2017 | Retrospective, Multicenter | 110 (90 ERCP, 10 EUS-CDS, 10 EUS-HGS) | EUS-BD + DS  Clinical Success: 95%  Similar patency of ERCP versus EUS-BD (better when metal stent was used). Higher AEs rate for EUS-BD. |
| Debourdeau A, 2018 | Retrospective, Multicenter | 31 | BD in patients with DS (11 EUS-HGS, 11 PTBD, 8 ERCP, 1 EUS-CDS)  In patients with same-session procedures, there were lower hospitalization time and lower AEs compared to 2-sessions management.  EUS-HGS tended to increased patency versus other modalities |
| Yamao K, 2018 | Retrospective, Multicenter | 39 | BD in patients with DS in place (overlaying the papilla in 59%).  EUS-BD versus ERCP:   - Technical Success: 95.2% versus 56%, p<0.01 (differences were even higher when the DS covered the papilla) - AEs: 43% vs 32%, p=0.65 |
| Mangiavillano M, 2021 | Retrospective, Multicenter | 23 | EUS-CDS (60% EUS-GBD) through the meshes of DS  Technical success: 96% |
| So H, 2021 | Retrospective, Single Center | 18 | EUS-antegrade stenting + DS  Technical success: 96%  AEs: 24%  Reinterventions: 8%  When compared with conventional EUS-BD, antegrade stenting showed higher patency. |
| **EUS-GE + EUS-BD** | | | |
| **Study** | **Design** | **Patients** | **Results** |
| Canakis A, 2021 | Retrospective, Single Center | 23 | Single-session EUS-GE + EUS-HGS  Technical Success: 96% + 100%  Clinical Success: 100% + 73%  AEs: 22%  Reinterventions: 0% + 14% |
| Bronswijk M, 2023 | Retrospective, Multicenter | 101 | Same-session EUS-GE + EUS-BD, compared with 53 surgical double bypass  TS: 96% vs 100%, p=0.1  CS: 94% vs 82%, p=0.05  AEs: 11% vs 35%, p=0.002 |
| **General** | | | |
| **Study** | **Design** | **Patients** | **Results** |
| Vanella G, 2022 | Retrospective, Multicenter | 93 | Outcomes of 6 endoscopic combinations including DS, EUS-GE, ERCP, EUS-CDS, EUS-HGS  Significantly different rate of dysfunction (p=0.009):   - EUS-GE+HGS: 0% - EUS-GE+TPS: 18% - EUS-GE+CDS: 31% - DS+TPS: 53% - DS+CDS: 83% |
| Rizzo GEM, 2023 | Systematic Review | 11 studies | EUS-BD in double obstruction  Technical Success: 96%  Clinical Success: 85%  AEs: 29% |
| ERCP: Endoscopic Retrograde Cholangiopancreatography; DS: Duodenal Stent; EUS-BD: EUS-guided Biliary Drainage; PTBD: Percutaneous Transhepatic Biliary Drainage; BD : Biliary Drainage; EUS-CDS: EUS-Choledochoduodenostomy; EUS-HGS: EUS-Hepaticogastrostomy; EUS-GBD: EUS-gallbladder drainage; EUS-GE: EUS-Gastroenterostomy; TPS: transpapillay metal stent | | | |

**Supplementary Table 10**

| **First Author/year** | **Journal** | **Number of studies** | **RCT included** | **Prospective** | **Sample size** | **EUS-CDS** | **EUS-HGS** | **Specialty** |
| --- | --- | --- | --- | --- | --- | --- | --- | --- |
| Mao et al. 2010 | SLEPT | 13 | 1 (7.7%) | 4 (30.7%) | 759 | 359(47.2%) | 400 (52.8%) | HPB Surgeon |
| Uemura et al. 2017 | JCG | 10 | 1 (10%) | 4 (40%) | 434 | 226 (51.1%) | 208 (47.9%) | Gastroenterology |
| Hedjoudje et al. 2019 | UEG | 5 | 1 (20%) | 3 (60%) | 377 | 173 (45.8%) | 204 (54.2%) | Gastroenterology |

Legend: JCG= Journal of Clinical Gastroenterology; UEG= United European Gastroenterology Journal; SLEPT= Journal of Surgical & Laparoscopic Endoscopic Percutaneous Techniques

**Supplementary Table 11**

| Outcomes of interest | No. of studies | OR (95% CI)  EUS-CDS vs EUS-HGS | P-value | | C-Q, I^2^ (%) | | P-value for reporting bias ^ | | | |
| --- | --- | --- | --- | --- | --- | --- | --- | --- | --- | --- |
|  |  |  |  |  |  |  | **Egger** | **Begg** | | |
| Clincal succes | 3 | 0.96 (0.69 to 1.32) | 0.784 | 0.902; 0 | | 0.124 | | | 0.117 |  |
| Technical Succes | 3 | 0.87 (0.70 to 1.07) | 0.181 | 0.880; 0 | | 0.210 | | | 0.601 |  |
| Adverse events | 3 | 0.85 (0.67 to 1.08) | 0.177 | 0.266 24 | | 0.251 | | | 0.601 |  |

**Legend:** EUS-CDS=Endoscopic ultrasound-guided choledochoduodenostomy; EUS-HGS= Endoscopic ultrasound-guided hepaticogastrostomy; OR= Odds Ratio; CI= Confidence interval; C-Q= P-value of Cochran’s test; I^2^= Higgins test; ^=A reporting bias non-negligible is considered for P values <0.10.

**Supplementary Table 12**

| **Author, yr** | **Study type, centers** | **Studies included (tot)** | **RCT included** | **N° Pts (tot)** | **Intervention (n)** | **Compared method (n)** | **Comment** |
| --- | --- | --- | --- | --- | --- | --- | --- |
| **Bang, 2018** | RCT | - | - | 67 | EUS-BD (33) | ERCP (34) | Similar rates of adverse events and outcomes. EUS-BD is a viable alternative to ERCP |
| **Fabbri, 2019** | Case series, single | - | - | 5 | EUS-CDS with ECE-LAMS (5) | - | EUS-CDS with ECE-LAMS doesn’t affect subsequent surgery |
| **Gajoux, 2021** | Retrospective, multicenter | - | - | 21 | EUS-CDS with ECE-LAMS (21) | - | EUS-CDS is technically feasible with acceptable post-operative outcomes |
| **Janet, 2023** | Retrospective, multicenter | - | - | 156 | ERCP (128) | EUS-CDS with ECE-LAMS (28) | EUS-CDS is technically easier with less post-operative complication and no difference in oncological outcomes |

**Supplementary Table 13**

| Billroth 2 reconstruction | | | | | | | |  |
| --- | --- | --- | --- | --- | --- | --- | --- | --- |
| Study | Design | | Patient/period | Results1 | Results2 | | |  |
| Kim (1) | | Propensity score match study | 110 patients  2004/2020 | Bile duct cannulation and complete CBD stone removal were not significantly different between the SE and CE groups | Among adverse events, the rate of post-ERCP pancreatitis showed higher tendency in the CE group than in the SE group | | |  |
| Park (3) | | Meta-analyses | 25 studies  2446 patients | Afferent loop intubation rate was higher  with the forward-viewing endoscope (90.3%, vs. 86.8%) | Cannulation rate was higher with the side-viewing endoscope (92.3% vs. 91.1%) | Bowel perforation rate was higher with the side-viewing endoscope (3.6% vs. 3.0%) | Pancreatitis rate and bleeding rate were higher with the forward-viewing endoscope (5.4%, vs.  2.5%).  (3.0%, vs. 2.0%,) |  |
| Iwashita (4) | | Retrospective cohort study | 64 pts 2007/2019 | The adverse event rate was 11.4% in eus BD vs 27.6% in PTBD (*P* = .119). | | | | |
| Sharaiha (5) | | Meta-analyses | Nine studies with 483 patients | EUS-BD was associated with better clinical  success (OR, .45), fewer post procedure adverse events (OR, .23), and lower rate of reintervention (OR, .13) | | | | |

| Roux an Y reconstruction | | | | | |
| --- | --- | --- | --- | --- | --- |
| Study | Design | Patients period | Results1 | Results2 | Results3 |
| Khashab(6) | multicenter comparative cohort study | 98 patients  2008/2014 | EUS BD technical success was  achieved in 98 % vs  65.3 % patients in the  e-ERCP group (OR 12.48, P=0.001) | Clinical success  was attained in 88% of patients in EUS-BD  group as compared to 59.1% in the e-ERCP group  (OR 2.83, P=0.03). | AEs occurred more commonly  in the EUS-BD group (20% vs. 4%, P=0.01).  90 % of AEs were mild/moderate. |
| Minaga (7) | multicenter prospective study | 40 patients  2016/2018 | Technical success rate was 100%  Clinical success rate was 95% | Early AEs were noted in 15% of patients Late AEs occurred in 15% of patients | |
| Ogura (8) | Multicenter prospective study | 49 patients 2014/2016 | EUS-HGAS was successfully carried out in 40 patients (technical  success rate: 85.7%)  Adverse events were seen in 10.2% (5/49) of cases. | | |

| Gastric by-pass reconstruction | | | | | |
| --- | --- | --- | --- | --- | --- |
| Study | Design | Patient period | Results1 | Results2 | Results3 |
| Deliwala (9) | Meta-analysis | 16 studies (470 patients) | technical success (TS) rate was 96%  and clinical success was 91% | EDGE TS was comparable to LA-ERCP (97% vs. 98%; *p* = *0.95*) and E-ERCP (100% vs. 66%; p = *0.06*)*.* | Pooled rate of all adverse events with EDGE was 17%  (14–24.6, *I*2 = 32%  EDGE procedure time and hospital stay were shorter than LA-ERCP and E-ERCP  (*p* < 0.001). |
| Connell (10) | Meta-analysis | 53 studies involving 857 patients | LAERCP and EDGE had high rates of success in the management of CDL post-RYGB. LAERCP had fewer  complications but was associated with longer procedure times. BAE had lower success rates than both LAERCP and EDGE. | | |
| Runge (12) | multicenter retrospective | 178 patients  2015/2019 | Malignant biliary stricture in 4% of patients | | |
| Wang (13) | Retrospective study | 130 patients  2009 2019 | Malignant biliary stricture in 8% of patients | | |
