# Supplementary material for: Biliary drainage in patients with malignant distal biliary obstruction: results of an Italian consensus conference
Source: Surg Endosc. 2024 Sep 25;38(11):6207–26. doi: 10.1007/s00464-024-11245-4 (PMC11525304; doi:10.1007/s00464-024-11245-4)
Supplement: Supplementary file 5 — Supplementary file5 (DOCX 23 KB) [file 464_2024_11245_MOESM5_ESM.docx]

**Supplementary Table 14:** literature search criteria

| ***Statement*** | ***Search criteria*** |
| --- | --- |
| **Statement #1**  **i-EUS group suggests EUS choledocho-duodenostomy (EUS-CDS) as an alternative to ERCP as first attempt for biliary drainage in patients with unresectable distal malignant biliary obstruction.**  *(Level of evidence: 2, grade of recommendation: strong)* | (((((((((ercp[MeSH Terms]) OR (cholangiopancreatographies, endoscopic retrograde[MeSH Terms])) AND (malignant distal biliary obstruction[Title/Abstract])) OR (DMBO[Title/Abstract])) OR (malignant distal biliary stenosis[Title/Abstract])) OR (biliary drainage[Title/Abstract])) AND (biliary tract diseases[MeSH Terms])) AND (english[Language])) |
| **Statement #2a**  **-EUS group recommends against routine preoperative biliary drainage in patients with resectable malignant distal biliary obstruction.** *(Level of evidence: 2, grade of recommendation: strong)* | (((ERCP) OR (biliary drainage)) AND (preoperative) AND ((distal malignant biliary obstruction) OR (resectable malignant biliary obstruction) OR (resectable distal malignant biliary obstruction) OR (resectable pancreatic cancer))) NOT ((advanced) OR (palliative) OR (palliation) OR (unresectable) OR (hilar) OR (perihilar) OR (peri-hilar))) |
| **Statement #2b**  **i-EUS group recommends preoperative biliary drainage in patients with cholangitis, severe jaundice, in those planned for neoadjuvant therapy, and when a delay in surgery is anticipate.**  *(Level of evidence: 1, grade of recommendation: strong)* | (((ERCP) OR (biliary drainage)) AND (preoperative) AND ((distal malignant biliary obstruction) OR (resectable malignant biliary obstruction) OR (resectable distal malignant biliary obstruction) OR (resectable pancreatic cancer))) NOT ((advanced) OR (palliative) OR (palliation) OR (unresectable) OR (hilar) OR (perihilar) OR (peri-hilar))) |
| **Statement #3**  ***i-EUS group recommends EUS-guided choledochoduodenostomy (EUS-CDS) over PTBD after failed ERCP in malignant unresectable distal MDBO.***  *(Level of evidence: 1, grade of recommendation: strong)* | (((((((((ercp[MeSH Terms]) OR (cholangiopancreatographies, endoscopic retrograde[MeSH Terms])) AND (malignant distal biliary obstruction[Title/Abstract])) OR (DMBO[Title/Abstract])) OR (malignant distal biliary stenosis[Title/Abstract])) OR (biliary drainage[Title/Abstract])) AND (biliary tract diseases[MeSH Terms])) AND (english[Language])) |
| **Statement #4**  **i-EUS group suggests performing EUS-BD under deep sedation or general anaesthesia; the choice between these two options should be based on patient clinical condition and anaesthesiologist preference.**  *(Level of evidence: 4, grade of recommendation: weak)* | ((((((malignant biliary obstruction) OR (biliary drainage)) OR (ERCP)) OR (endoscopic ultrasound)) OR (EUS)) AND (sedation) |
| **Statement #5**  **-EUS recommends EUS-choledocoduodenostomy for the treatment of MBDO after ERCP failure. EUS-guided gallbladder drainage (EUS-GBD) and EUS-guided heaticogastrostomy (EUS-HGS) should be considered in case of unapproachable EUS-CDS.**  *(Level of evidence 2; Grade of recommendation: strong)* | (((((("malignant biliary obstruction"[All Fields]) OR (MBO)) OR (biliary obstruction)) AND (distal)) AND ((((("ercp"[All Fields]) OR (endoscopic retrograde cholangio-pancreatography)) OR ("endoscopic retrograde cholangiogram"[All Fields])) OR ("endoscopic retrograde cholangiopancreatography"[All Fields])) AND ("failure"[All Fields]))) AND ((EUS) OR (Endoscopic Ultrasound) OR (choledocoduodenostomy) OR (hepaticogastrostomy) OR (gallbladder drainage) OR (rendezvous))) AND ((drainage) OR (biliary drainage)) |
| **Statement #6**  **i-EUS group states that EUS-guided gallbladder drainage (EUS-GBD) may be considered a rescue strategy for biliary drainage in unresectable patients with MBDO and patent cystic duct when other EUS-BD approaches are not feasible.**  *(Level of evidence 3; Grade of recommendation: weak)* | (("malignant biliary obstruction"[All Fields]) OR (MBO) OR (biliary obstruction)) AND ((EUS) OR (Endoscopic Ultrasound) OR (gallbladder drainage) OR (EUS-GBD)) |
| **Statement #7a**  **iEUS recommends the use of either self-expandable metal stents (SEMS) or lumen-apposing metal stents (LAMS) for EUS-guided choledochoduodenostomy (EUS-CDS).**  *(Level of evidence 3; Grade of recommendation: strong)* | ((choledochoduodenostomy) OR (biliary drainage) OR (malignant biliary obstruction) OR (EUS-guided) OR (failed ERCP) OR (LAMS) OR (stent) AND (english[Language]) AND human AND [SEMS OR LAMS] |
| **Statement #7b**  **iEUS suggests that of electrocautery enhanced LAMS might be preferred in patients with dilated (≥ 15 mm) common bile duct since a slight reduction in pooled incidence of adverse events was observed.**  *(Level of evidence 3; Grade of recommendation: weak)* | ((choledochoduodenostomy) OR (biliary drainage) OR (malignant biliary obstruction) OR (EUS-guided) OR (failed ERCP) OR (LAMS) OR (stent) AND (english[Language]) AND human AND [SEMS OR LAMS] |
| **Statement #8**  **I-EUS does not suggest the use of 6 x 8 mm over 8 x 8 mm LAMS since no advantages has been demonstrated; 6 x 8 mm LAMS could be used in case of small CBD diameter.**  *(Level of evidence 4; Grade of recommendation: weak)* | (("malignant biliary obstruction"[All Fields]) OR (MBO) OR (biliary obstruction)) AND ((EUS) OR (Endoscopic Ultrasound) OR (choledochoduodenostomy) OR (EUS-CDS)) AND ((LAMS) OR (lumen apposing metal stent)) |
| **Statement #9**  **There is no evidence to suggest in favor or against double pig-tail plastic stents (DPPS) placement through the LAMS in patients undergoing EUS-guided choledochoduodenostomy (EUS-CDS). i-EUS states that DPPS placement could be considered in selected cases.** *(Level of evidence 4; Grade of recommendation: weak)* | (("malignant biliary obstruction"[All Fields]) OR (MBO) OR (biliary obstruction)) AND ((EUS) OR (Endoscopic Ultrasound) OR (choledochoduodenostomy) OR (EUS-CDS)) AND ((LAMS) OR (lumen apposing metal stent)) |
| **Statement #10**  **i-EUS suggests the use of dedicated stents in patients undergoing EUS-guided heaticogastrostomy (EUS-HGS) for MBDO.**  *(Level of evidence 5, Grade of recommendation: open)* | (("malignant biliary obstruction"[All Fields]) OR (MBO) OR (biliary obstruction)) AND ((EUS) OR (Endoscopic Ultrasound) OR (hepaticogastrostomy) OR (EUS-HGS)) |
| **Statement #11**  **i-EUS suggests that EUS-gastroenterostomy (EUS-GE) may be preferred over Enteral Stenting in patients with malignant Gastric Outlet Obstruction.**  (*Level of evidence 3, Grade of recommendation: weak*) | Patient/population, Medical condition: “malignant gastric outlet obstruction”; Intervention: “EUS gastroenterostomy”; compare to: “enteral stenting”; outcome: NULL |
| **Statement #12a**  **i-EUS suggests that, in the setting of double obstruction, ERCP may be attempted whenever the papilla is reachable (especially in type 1 or 3 stenosis) or in case of previously placed duodenal stent.**  (*Level of evidence 4, Grade of recommendation: weak*) | (((("biliary"[Title/Abstract] OR "bile"[Title/Abstract] OR "choledochus"[Title/Abstract]) AND ("jaundice"[Title/Abstract] OR "obstruction"[Title/Abstract] OR "stenosis"[Title/Abstract] OR "stricture"[Title/Abstract])) AND ("gastric outlet"[Title/Abstract] OR "duodenal stenosis"[Title/Abstract] OR "duodenal obstruction"[Title/Abstract] OR "duodenal stent"[Title/Abstract] OR "enteral stent"[Title/Abstract])) AND ("malignan*"[Title/Abstract] OR "neoplas*"[Title/Abstract] OR "tumor"[Title/Abstract] OR "cancer"[Title/Abstract])) AND ("endoscop*"[Title/Abstract] OR "EUS"[Title/Abstract] OR "ERCP"[Title/Abstract]) |
| **Statement #12b**  **i-EUS suggests that, in naïve patients with double obstruction, EUS-guided double bypass may be considered where adequate expertise is available.**  (*Level of evidence 4, Grade of recommendation: weak*) |  |
| **Statement #13**  **i-EUS suggests that, in the specific setting of double obstruction, EUS-guided heaticogastrostomy (EUS-HGS) may be favored over EUS-guided choledochoduodenostomy (EUS-CDS) due to longer stent patency.**  (*Level of evidence 4, Grade of recommendation: weak*) | (("Constriction, Pathologic"[Mesh] OR "Duodenal Obstruction"[Mesh] OR "Gastric Outlet Obstruction"[Mesh] OR "Pyloric Stenosis"[Mesh] OR “duodenal stenosis”[tw] OR “duodenal obstruction”[tw] OR “pyloric stenosis”[tw] OR “pyloric obstruction”[tw] OR “gastric stenosis”[tw] OR “gastric obstruction”[tw] OR “gastric outlet”[tw]) AND ("Cholestasis"[Mesh] OR "Bile Duct Diseases"[Mesh] OR "Bile Ducts"[Mesh] OR cholesta*[tw] OR “bile duct obstruction”[tw] OR “bile duct compression”[tw] OR “bile duct stenosis”[tw] OR “biliary obstruction”[tw] OR “biliary compression”[tw] OR “biliary stenosis”[tw]) AND ("Neoplasms"[Mesh] OR malignan*[tw] OR neoplas*[tw] OR cancer[tw] OR tumor[tw]) AND "Endosonography"[Mesh] OR "Endoscopy, Digestive System"[Mesh] OR EUS[tw] OR endosonography[tw] OR “endoscopic ultra sound”[tw] OR endoscop*[tw] AND "Drainage"[Mesh] OR drain*[tw] OR “hepaticogastrostomy” [tw] OR choledocoduodenostomy” [tw])) |
| **Statement #14**  **i-EUS suggests either ERCP or EUS-guided choledochoduodenostomy (EUS-CDS) as the first line treatment of resectable MBDO.**  (*Level of evidence 4, Grade of recommendation: weak*) | ((ERCP[Title/Abstract] OR "Endoscopic Retrograde"[Title/Abstract]) AND (EUS[Title/Abstract] OR Endosonography[Title/Abstract] OR "Endoscopic Ultrasound"[Title/Abstract])) AND (surgery[Title/Abstract] OR surgical[Title/Abstract] OR neoadjuvant[Title/Abstract]) |
| **Statement 15a**  **In patient with malignant distal biliary stenosis and surgical altered anatomy, treatment choice depends on disease extension and type of reconstruction. i-EUS suggests to consider EUS-BD over laparoscopic-/enteroscopy-assisted ERCP and PTBD in patient with Roux-en-Y anatomy.**  (*Level of evidence 4, Grade of recommendation: weak*) | (("biliary"[Title/Abstract] OR "bile"[Title/Abstract]) AND ("obstruction"[Title/Abstract] OR "stenosis"[Title/Abstract] OR "stricture"[Title/Abstract])) AND ("altered anatomy"[Title/Abstract]) AND ("endoscop*"[Title/Abstract] OR "EUS"[Title/Abstract] OR "ERCP") AND ("malignan*"[Title/Abstract] OR "neoplas*"[Title/Abstract] OR "tumor" [Title/Abstract] OR "cancer"[Title/Abstract])) |
| **Statement 15b**  **i-EUS suggests to consider EUS-BD as a rescue strategy after failed ERCP with lateral-viewing or cap-assisted frontal-viewing endoscopes in patients with Billroth II reconstruction.**  (*Level of evidence 4, Grade of recommendation: weak*) |  |
| **Statement #16**  **I-EUS suggests multidisciplinary discussion of patients with distal malignant obstruction in whom the biliary drainage could impact on the main outcomes, in particular patients with resectable cancer, altered anatomy and double obstruction.**  (*Level of evidence 5, Grade of recommendation: weak*) | <EUS-guided biliary drainage AND multidisciplinary><therapeutic endoscopic ultrasound AND multidisciplinary><EUS-guided biliary drainage AND setting> <therapeutic endoscopic ultrasound AND setting> |
| **Statement #17**  **I-EUS suggests endoscopic biliary drainage to be performed in a setting, where adequate competencies in interventional bilio-pancreatic management are available.**  (*Level of evidence 5, Grade of recommendation: weak*) | <EUS-guided biliary drainage AND setting> <therapeutic endoscopic ultrasound AND setting><EUS-guided biliary drainage AND management> <therapeutic endoscopic ultrasound AND management> |
| **Statement #18**  **i-EUS does not suggest any specific diet after EUS-guided biliary drainage to prevent stent disfunction.**  (*Level of evidence 5, Grade of recommendation: weak*) | (choledochoduodenostomy) OR (biliary drainage) OR (ERCP) OR (EUS-guided) OR (LAMS) AND ((food-impaction) OR (LAMS dysfunction) OR (diet)) |
| **Statement #19**  **i-EUS does not suggest the routine use of antibiotic prophylaxis, to reduce the risk of post-procedural complications after EUS-guided biliary drainage.**  **i-EUS suggest that antibiotic prophylaxis should be offered in selected patients (e.g., immunocompromised patients, expected incomplete biliary drainage).**  (*Level of evidence 5, Grade of recommendation: weak*) | (((biliary drainage[tiab]) OR (ercp[tiab]) OR (eus guided biliary drainage[tiab])) AND ((medical therapy[tiab]) OR (drug therapy[tiab]) OR (antibiotics[tiab])) AND ((Complication[tiab]) OR (Infection[tiab]) OR (sepsis[tiab]) OR (cholangitis[tiab]))) NOT ("case reports"[Publication Type]) NOT ("review"[Publication Type]) |
| **Statement #20**  **i-EUS does not suggest the administration of medical therapy to improve endoscopic outcomes after biliary drainage for malignant distal biliary obstructions.**  **iEUS does not suggest the use of ursodeoxycholic acid (UDCA) since it is not effective in preventing recurrent biliary obstruction after SEMS placement and may increase the risk of stent occlusion.**  (*Level of evidence 5, Grade of recommendation: weak*) | ((biliary drainage[tiab]) OR (ercp[tiab]) OR (eus guided biliary drainage[tiab])) AND ((medical therapy[tiab]) OR (drug therapy[tiab]) OR (ursodeoxycholic acid[tiab])) AND ((Complication) OR (obstruction) OR (patency) OR (displacement))) NOT ("case reports"[Publication Type]) NOT ("review"[Publication Type]) |
| **Statement #21**  **i-EUS suggests obtaining a “goal-based” informed consent for endoscopic malignant biliary drainage prior to either EUS-guided drainage or conventional ERCP.**  *(Level of evidence: 5; Grade of recommendation: weak)* | (("informed consent"[MeSH Terms] OR ("informed"[All Fields] AND "consent"[All Fields]) OR "informed consent"[All Fields]) AND ("biliary"[All Fields] AND ("drainage"[MeSH Terms] OR "drainage"[All Fields] OR "drainaged"[All Fields] OR "drainages"[All Fields]))) OR (("informed consent"[MeSH Terms] OR ("informed"[All Fields] AND "consent"[All Fields]) OR "informed consent"[All Fields]) AND (("endoscope s"[All Fields] OR "endoscoped"[All Fields] OR "endoscopes"[MeSH Terms] OR "endoscopes"[All Fields] OR "endoscope"[All Fields] OR "endoscopical"[All Fields] OR "endoscopically"[All Fields] OR "endoscopy"[MeSH Terms] OR "endoscopy"[All Fields] OR "endoscopic"[All Fields]) AND "biliary"[All Fields] AND ("drainage"[MeSH Terms] OR "drainage"[All Fields] OR "drainaged"[All Fields] OR "drainages"[All Fields]))) OR (("informed consent"[MeSH Terms] OR ("informed"[All Fields] AND "consent"[All Fields]) OR "informed consent"[All Fields]) AND ("endoscopie"[All Fields] OR "endoscopy"[MeSH Terms] OR "endoscopy"[All Fields] OR "endoscopies"[All Fields] OR "endoscopy s"[All Fields])) |
